# Fluid dynamics of respiratory droplets in the context of COVID-19: Airborne and surfaceborne transmissions

**DOI:** 10.1063/5.0063475

**Published:** 2021-08-18

**Authors:** Pallavi Katre, Sayak Banerjee, Saravanan Balusamy, Kirti Chandra Sahu

**Affiliations:** 1 Department of Chemical Engineering, Indian Institute of Technology Hyderabad, Sangareddy 502285, Telangana, India; 2 Department of Mechanical and Aerospace Engineering, Indian Institute of Technology Hyderabad, Sangareddy 502285, Telangana, India

## Abstract

The World Health Organization has declared COVID-19 a global pandemic. Several countries have experienced repeated periods of major spreading over the last two years. Many people have lost their lives, employment, and the socioeconomic situation has been severely impacted. Thus, it is considered to be one of the major health and economic disasters in modern history. Over the last two years, several researchers have contributed significantly to the study of droplet formation, transmission, and lifetime in the context of understanding the spread of such respiratory infections from a fluid dynamics perspective. The current review emphasizes the numerous ways in which fluid dynamics aids in the comprehension of these aspects. The biology of the virus, as well as other statistical studies to forecast the pandemic, is significant, but they are not included in this review.

## I. INTRODUCTION

**FIG. 1.**
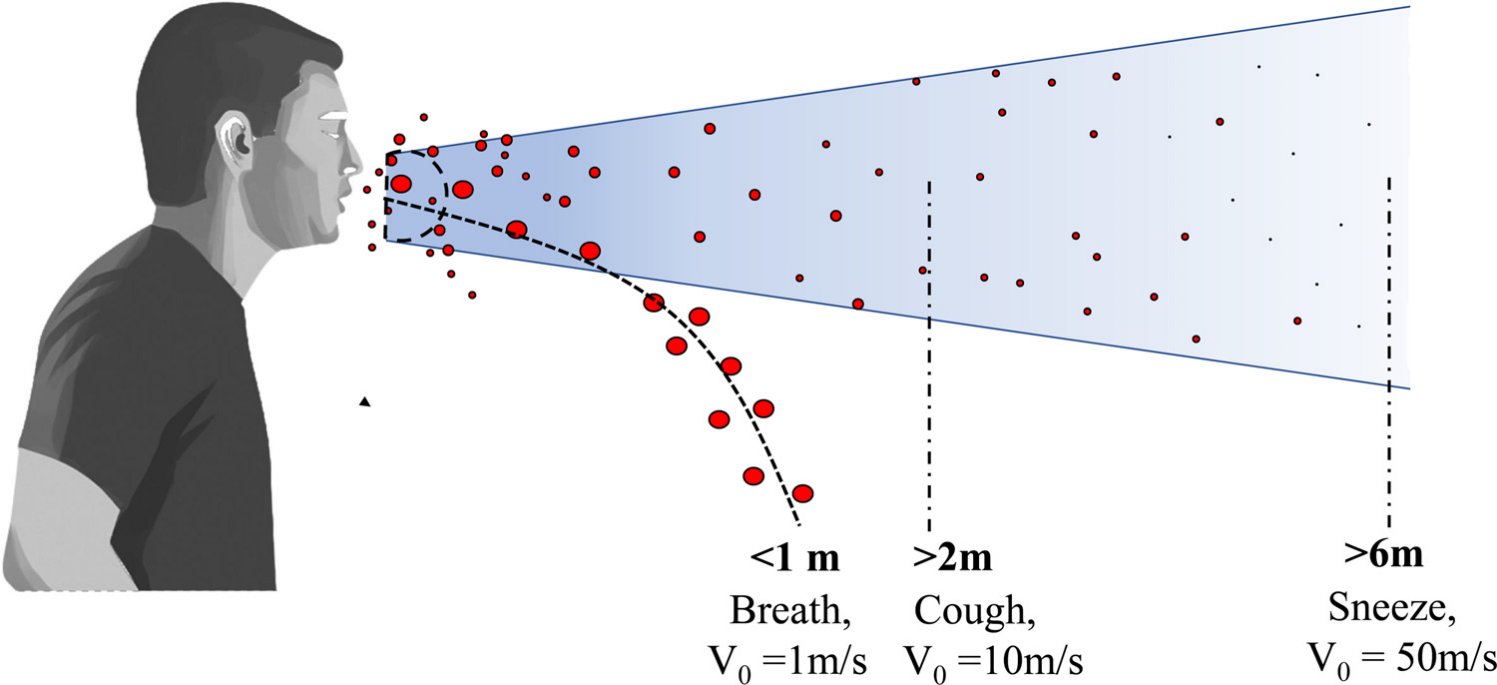
Schematic of droplet transmission ejected during sneezing and coughing. A typical conical jet flow of droplets during sneezing and coughing has a cone angle in the range of $22^{{}^{\circ}}-28^{{}^{\circ}}$.

Respiratory infections are spread by virus-containing droplets and aerosols exhaled by infected individuals during breathing, speaking, coughing, and sneezing.^1,2^ The fluid lining of the respiratory tract is thought to be the source of respiratory droplets.^3^ Respiratory droplets of an infected person contain water, virus, and several other ingredients. By conducting physicochemical characterization, the concentrations of salt, mucin, and surfactant per liter of saliva solution were reported to be 9, 3, and 0.5 g/l, respectively.^4^ The number of droplets, their size, and the velocity of the droplets passing through the respiratory tracts affect the transmission.^5^ While sneezing produces approximately 10^4^ number of droplets that flow at a speed of up to 20 m/s, coughing produces 10–100 times fewer droplets that move at around 10 m/s. A human talking also produces about 50 particles/s.^6^ In a confined room, if one infected person speaks without using a mask, 2.5 virions are inhaled per minute.^7^ Droplets ejected from sneezing and coughing have different ejection velocities and the forces like diffusion, drag, and gravity affect the droplet motion.^8^ Droplets are ejected in the form of conical jet flow having cone angle in the range of $22^{{}^{\circ}}-28^{{}^{\circ}}$.^9,10^ A schematic representation of droplet transmission ejected during sneezing and coughing is shown in Fig. 1. SARS-CoV-2 virus possesses aerosol and surface stability (the virus remains alive and infectious in aerosol for hours); therefore, airborne transmission can coexist with the close-contact transmission.^11^ Furthermore, environmental factors such as ambient temperature and humidity have an impact on droplet transmission.^12^

Droplets are categorized into two types by the medical infectious disease community, namely, respiratory droplets and aerosols. Droplets greater than 5–10 μm in diameter are classified as respiratory droplets, whereas droplets smaller than 5 *μ*m are classified as aerosols.^13^ The two modes of transmission of the virus causing COVID-19 are (1) airborne transmission and (2) surfaceborne transmission. This article mainly discussed the role of fluid dynamics to understand these transmissions.

## II. AIRBORNE TRANSMISSION

Several studies on coronavirus (SARS-CoV-2) support aerosol transmission.^14–18^ The previous investigations on airborne transmission are listed in Table I. Recent investigations show that airborne transmission plays a dominant role in the disease's rapid spread in several counties. The overall airborne transmission process after ejection of the respiratory droplets is briefly summarized before highlighting some of the key findings of the recent studies. The virus particles are captured by the saliva and mucus droplets that are ejected from the nose and mouth of an infected person during a coughing or sneezing event. Even talking and passive breathing can contribute to a continuous dispersion of the viral particles in the environment.^19,20^ The subsequent spread of the viral particles intimately depends on the trajectory of these aerosolized droplets through the air, and it is here that fluid dynamics play a crucial role. At the time of the ejection, the droplets are embedded in the puff of expelled air volume that is moving at a coherent velocity with respect to the ambient air. The volume of this puff containing the aerosolized droplets increase through entrainment processes and this is attenuated by a rapid decrease in the puff velocity. The larger droplets however have greater inertial momentum and separate out of the puff. The larger droplets are also affected by gravity and tend to fall and settle on nearby surfaces quicker (see Fig. 1). In contrast, the smaller droplets tend to remain within the puff, with their velocities decelerating in conjunction with the exhaled puff. Evaporation processes decrease the volume of the aerosolized droplets with time until a nonevaporating core of nonvolatile organic liquids and salt shells is left. Eventually, the puff loses coherence and the droplet cores disperse freely into the turbulent ambient air stream. Thus a series of important physical and fluid dynamical processes govern the evolution of the viral-laden droplets post ejection. These include the initial velocity of ejection (cough or sneeze or breathing), the initial droplet size distribution, the trajectory of the larger sized droplets through the air, the fluid dynamic evolution of the puff containing the smaller aerosolized droplets, the rate of evaporation, important meteorological parameters like humidity, temperature, and wind velocity. This section takes a comprehensive look at all of these factors.

**TABLE I.** A list of previous studies on airborne transmission.

References	Remarks
Morawska and Cao^14^	Study supporting airborne transmission of COVID-19
Morawska and Milton^15^	Study supporting airborne transmission of COVID-19
Scheuch^16^	Role of breathing in airborne transmission of COVID-19
Bahl^17^	A review supporting aerosol transmission of COVID-19
Setti *et al.*^18^	Airborne transmission and social distancing
Yang *et al.*^19^	Aerosol transport through speaking
Hossain and Faisal^20^	Investigation of aerosol cloud flow during coughing, talking and breathing
Xie *et al.*^21^	Investigation of the evaporation and movement of droplets expelled during respiratory activities
Duguid *et al.*^22^	Droplet size distribution for coughing, sneezing, and speaking
Chao *et al.*^23^	Droplet size distribution for coughing and speaking
Loudon and Roberts^24^	Droplet size distribution for coughing and talking
Morawska *et al.*^25^	Droplet size distribution all respiratory activities
Bourouiba *et al.*^26^	Droplet size distribution for coughing and sneezing
Dbouk and Drikakis^27^	Investigation of transport, dispersion, and evaporation of saliva particles arising from a human cough
Agrawal and Bhardwaj^28^	Analysis of the fluid dynamics and thermodynamics of the cough cloud
Bourouiba^29^	Demonstration of exhalations, sneezes, and cough clouds
Cummins *et al.*^30^	Mathematical modeling to study the dynamics of spherical droplets in the source-sink flows
Diwan *et al.*^31^	Transmission dynamics of sneeze/cough flow numerically
Li *et al.*^32^	Investigation of effect of environmental conditions on cough droplets in outdoor environment
Feng *et al.*^33^	Influence of wind and relative humidity on social distancing
Mittal *et al.*^34^	Estimation of airborne transmission risk with application of face mask and social distancing
Zhao *et al.*^35^	Effect of environmental conditions on the dispersion of respiratory droplets
Bar-On *et al.*^36^	Key-numbers of SARS-CoV-2 virus responsible for infection
Kumar^37^	The impact of weather conditions on the propagation of COVID-19 in India
Sun and Zhai^38^	Ventilation and social distancing effectiveness in preventive COVID-19 transmission
Zangmeister *et al.*^39^	Filtration efficiencies of different cloth mask materials
Akhtar *et al.*^40^	Prevention of airborne transmission using face mask and social distancing
Khosronejad *et al.*^41^	Face mask to suppress the airborne transmissions
Wei *et al.*^42^	Efficiency of various face mask and filter materials
Dbouk and Drikakis^43^	Prevention of respiratory droplet transmission using face mask

Droplets released during sneezing and coughing are affected by many forces such as diffusion, drag, and gravity force.^8^ The evolution of the droplet-laden exhaled puff trajectory is also affected by these forces. A theoretical Monte Carlo analysis of the impact of these forces on droplet evolution was conducted for the droplet sizes from 2.5 to 100 *μ*m and ejection velocity from 5 to 21 m/s ejected during sneezing and coughing.^8^ It was found that the small droplets remain suspended in the air for a long time whereas the bigger droplets travel a larger distance and fall on the ground as gravitational force dominates over diffusion and drag forces. A smaller-sized droplet having a radius of 2.5 *μ*m survives in the air for about 41 minutes as the effect of gravity is negligible for such droplets. In contrast, droplets of about 100 *μ*m size remain in the air only for 1.5 s. Figure 1 shows the exhalation and subsequent trajectory of respiratory droplets during sneezing and coughing at different velocities. It was found that large droplets released during coughing travel at an average velocity of 10 m/s and migrate a distance of more than 2 m, while those expelled during sneezing have a velocity of roughly 50 m/s and migrate more than 6 m. On the other hand, large droplets discharged when breathing move with velocity 1 m/s and travel a distance of less than 1 m.^21^

**FIG. 2.**
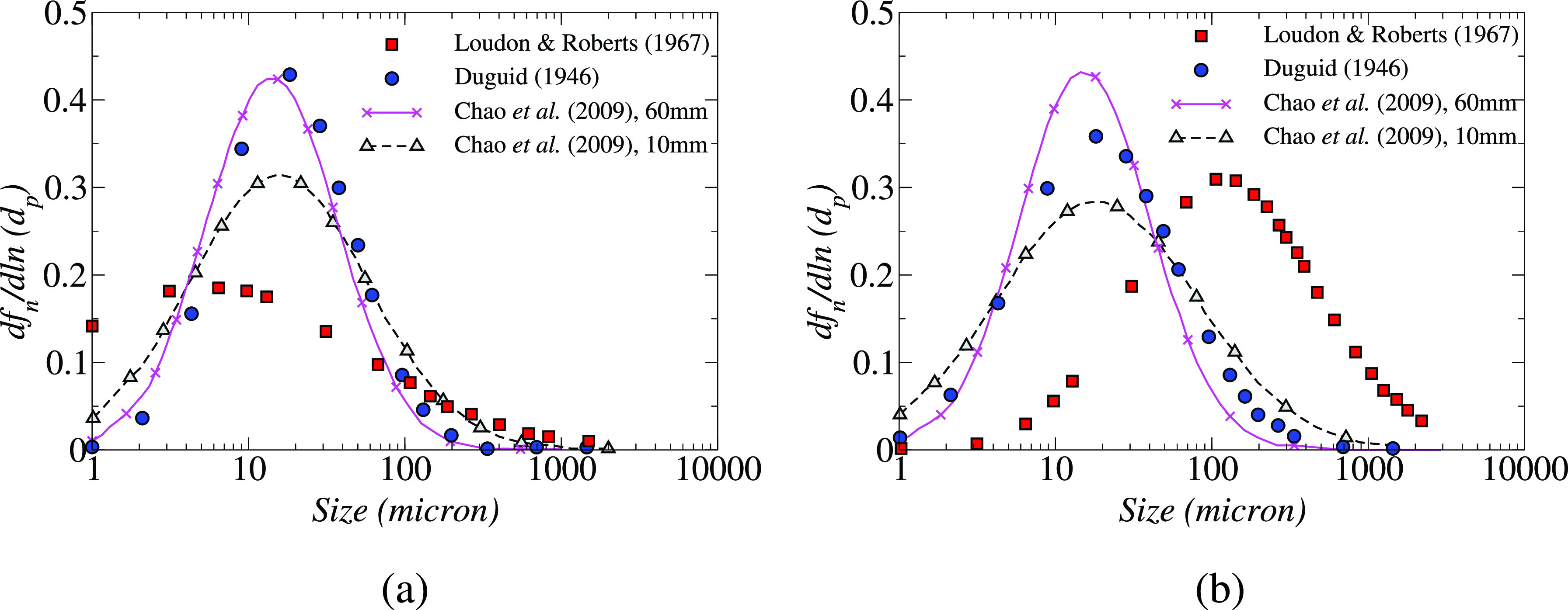
Droplet size distributions during (a) coughing and (b) speaking presented in different studies. Reproduced with permission from Chao *et al.*, “Characterization of expiration air jets and droplet size distributions immediately at the mouth opening,” J. Aerosol Sci. **40**(2), 122–133 (2009).^23^ Copyright 2009 Elsevier.

Experimental studies have been performed to investigate the droplet size distribution during different respiratory activities from a healthy person.^22–26^ Chao *et al.*^23^ used particle image velocimetry (PIV) and interferometric Mie imaging (IMI) techniques to measure the air velocity and droplet size during coughing and speaking. The coughing droplet count was calculated over 50 coughs, whereas the speaking droplet count was averaged over 10 counts of 1–100 at a distance of 10–60 mm from the mouth opening during coughing and speaking. The total number of ejected droplets was estimated based on the number of droplets observed in the measuring area. The estimates differ based on the method used, but the number of droplets at 10 mm distance was found to be around 150 droplets per liter of ejected air for a speaking event and 2400 droplets per liter of ejected air for a coughing event. The droplets were divided into 16 size classes based on Duguid's recommendations.^22^ During both coughing and sneezing, 6 *μ*m size class had the highest number count at the two measuring distances. Figure 2 depicts the droplet size profile fitted by log-normal distribution curve presented in terms of $df_{n}/d\;\text{ln}\;d_{p}$, where *f_n_* is the droplet number fraction and *d_p_* is the droplet diameter. The log-normal size distribution of the ejected droplets can be expressed as^11^

$$N_{e}(d_{p})=\frac{B}{d_{p}}\text{exp}\left[\frac{-{\left(\text{ln}\;\;d_{p}-\hat{\mu }\right)}^{2}}{2{\hat{\sigma }}^{2}}\right],$$ (1)

$\hat{\mu }$ and $\hat{\sigma }$ are the expected value and standard deviation of $\text{ln}\;(d_{p})$, which are also known as the geometric mean (GM) and the geometric standard deviation (GSD), respectively; *B* is a normalization constant. They observed that the geometric mean diameter was lesser during coughing (13.5 μm) as compared to that of speaking (16 μm) at a 10 mm distance from mouth opening. This observation indicates that the emission of air at a faster velocity while coughing may enhance the formation of smaller droplets than the lower exhalation velocity encountered when speaking. The droplet size distributions given by Loudon and Roberts^24^ and Duguid^22^ are also plotted in Fig. 2. It can be observed that the GM reported by Chao *et al.* and Duguid (1946) are fairly close, but it deviates significantly from the Loudon and Roberts (1967) data. The GM diameter reduces as the distance from the mouth opening is increased from 10 to 60 mm, this might be due to the shrinkage of droplets by evaporation.

**FIG. 3.**
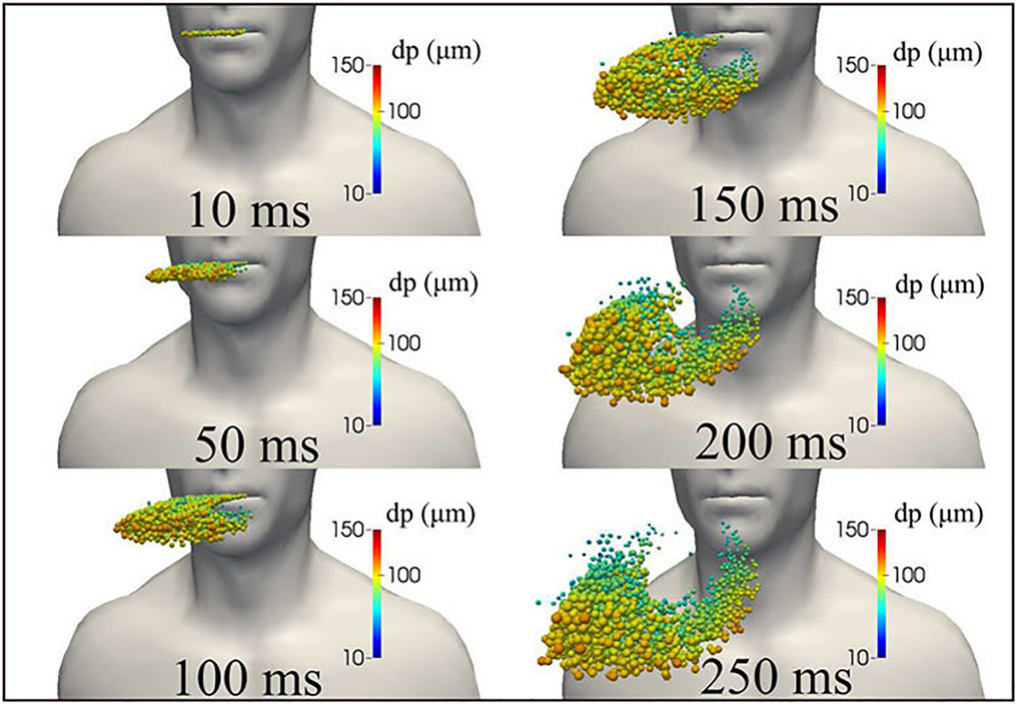
The kinematics of saliva droplet cloud released from human cough showing the diameter of the droplets. Because of gravity forces, larger droplets settle faster than smaller ones. The entire mass of ejected saliva is 7.7 mg, with a total number of droplets of 1008 in total. The temperature, pressure, and relative humidity in the environment are 20 °C, 1 atm, and 50%, respectively, with the ground temperature at 15 °C. The ambient air is considered to be stationary in this study. Reproduced with permission from T. Dbouk and D. Drikakis, “On coughing and airborne droplet transmission to humans,” Phys. Fluids **32**(5), 053310 (2020).^27^ Copyright 2020 AIP Publishing.

Dbouk *et al.*^27^ have numerically simulated the initial puff trajectory and droplet kinematics for a cough having an initial maximum ejection velocity of 8.5 m/s. A total time of 250 ms was simulated from the beginning of the coughing, and the mouth closure occurred at 120 ms (see Fig. 3). The ejected puff initially has a linear jet profile which expands and assumes a spheroidal shape at later times due to air entrainment. The initial trajectory of the droplet population for sizes from 10 to 120 *μ*m has also been simulated and it was found that the larger droplets settle down faster and move to the bottom of the ejected puff faster. Exhalations, sneezes, and coughs produce a multiphase turbulent gas cloud (a puff) that entrains ambient air and traps and transports clusters of droplets of various sizes. Droplets of different sizes from 2 to 1000 *μ*m are ejected during coughing. Exhaled droplets remain suspended in the air during the first 5–8 s after the cough event begins. The volume of infectious air is roughly 23 times greater than that evacuated by coughing.^28^ The cough cloud cools down to ambient temperature, although it remains somewhat moister than the surrounding air. Coughing into the elbow and using a handkerchief can both minimize the distance traveled by the cloud. The mask can dramatically limit the volume of infected air and the risk of infection to other people in the room. The pathogen-bearing droplets of various sizes in the gas cloud can travel 7–8 m for various combinations of an individual patient's physiology and environmental conditions, such as temperature and humidity.^29^ While the majority of the droplets remain trapped and clustered in the moving cloud, they infect the surfaces that settle along the pathway.

The influence of gravity on the varied sizes of spherical droplets present in a source-sink pair flow field was examined by Cummins *et al..*^30^ Droplets were divided into three categories based on their relative sizes: small, moderate, and large, and their behavior were examined in the presence and absence of gravity. As smaller droplets are more influenced by airflow, gravity may or may not affect the average travel time taken by the droplet from source to sink. The maximum horizontal distance traveled by intermediate size droplets ranges from a few *μ*m to a few hundred *μ*m during ordinary human respiration. Due to their greater inertia, larger droplets can travel longer from the source before being drawn into the sink, and their maximum traveled distance is calculated analytically. Diwan *et al.*^31^ proposed a similar droplet categorization and, by neglecting the thermodynamics effect, found that large droplets (> 100 *μ*m) can settle under gravity. For moderate-sized droplets (10–100 *μ*m) that evaporate, both gravitational settling and inertia are important. Small size droplets (<10 *μ*m) take part in thermodynamics by evaporating and follow the fluid streamline. This interesting and somewhat counterintuitive result can be explained by the fact that drag force scales with the diameter of the droplet, while gravity and inertial forces are volume-dependent (assuming the same density) and hence depend on the cube of the diameter. Thus, for large droplets, the drag force is insignificant and droplets approximate a ballistic trajectory. They have a large initial momentum, and hence, while their airborne time is short, they can travel fairly a large distance before dropping to the ground. At the small diameter spectrum, the drag force decreases linearly with diameter, but inertial and gravity forces decrease cubically with diameter. Hence, small droplets approximately follow the air and can travel a great distance with the air stream. For intermediate-sized droplets, the drag force and the gravity force are of the same order of magnitude. They are small enough to be decelerated by the drag force and large enough to feel the effect of gravity and fall away from the air stream.

Next, we review research that looked at the effects of climatic conditions, face masks, and social separation, as well as airborne transmission in both indoor and outdoor environments.

### A. Effect of meteorological parameters on social distancing

**FIG. 4.**
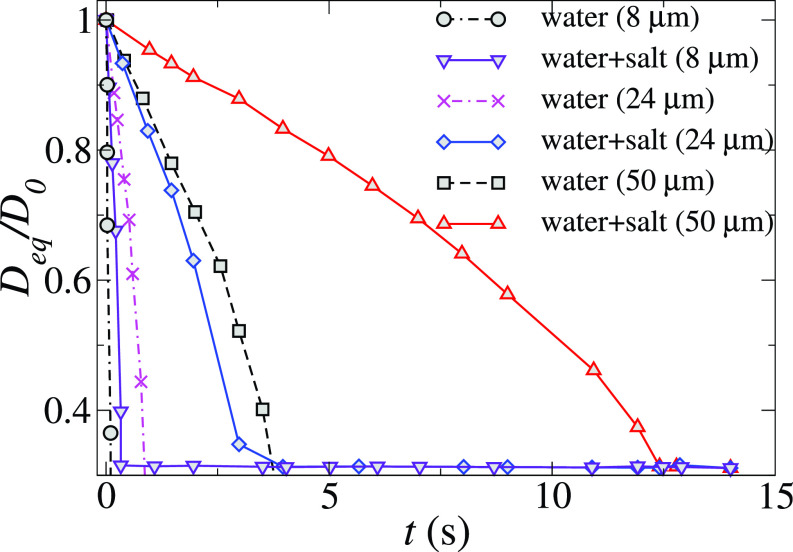
Comparison of the temporal variation of the normalized diameter of different initial size droplets of pure water with the water-salt solution. The initial temperature of the droplets is 36 °C. The values of the ambient temperature, RH, and wind speed are 30 °C, 0.84, and 2 m/s, respectively. Here, *D_eq_* and *D*_0_ represent the equivalent and initial diameter of the droplets, respectively. Reproduced with permission from Li *et al.*, “Dispersion of evaporating cough droplets in tropical outdoor environment,” Phys. Fluids **32**(11), 113301 (2020).^32^ Copyright 2020 AIP Publishing.

Meteorological parameters such as ambient temperature, relative humidity (RH), and wind flow affect the COVID-19 transmission and these have to be considered for ascertaining social distancing limit. Using a droplet tracking and evaporation model for people standing 1 and 2 meters away from the cougher, the effect of wind speed, relative humidity, and social distance on cough droplet evaporation and exposure in a tropical outdoor setting was examined.^32^ In this study, the effect of a nonvolatile component on droplet evaporation was taken into consideration by assuming that droplet evaporation is driven by the diffusion flux of droplet vapor into the air. The evaporation time for droplets having different sizes ejected during coughing is presented in Fig. 4. This figure also depicts the comparison of evaporation of droplets of pure water and salty water. It can be observed that the evaporation time of the droplet increases with an increase in droplet size due to the large volume to surface area ratio. Approximately, while half of these droplets remain suspended in the wake and are ultimately deposited on the cougher, 45% of these droplets sank quickly to the ground as a result of the downward velocity of the cough jet. The remaining 5% droplets exit the simulation domain at approximately *t *=* *15 s. Nonvolatile residues or droplet nuclei, which may be implicated in prolonged pathogen transmission in the air, are represented by the horizontal line in this figure. As shown in Fig. 4, these droplet nuclei are approximately 0.31 times the initial droplet diameters. The exact composition of the respiratory droplet is unclear, but salt is one of the important components of saliva. The addition of nonvolatiles like salt is observed to reduce the evaporation rate of the droplet. For 50 *μ*m droplets, the evaporation rate of the salty droplet is three times higher than the pure water droplets. The evaporation time of larger droplets is more affected by the addition of saltwater than the small size droplets. The downstream travel distance of the smaller droplets (of size ∼24 μm) exceeded the 8 m length of the study domain. The downstream travel distance of a 100 *μ*m droplet decreased to 6.6 m, while that of a 1000 *μ*m droplet was found to be 1.3 m. Since large droplets tend to settle quickly under the influence of gravity, rapid evaporation in drier climates may help to keep these large droplets airborne and increase their travel distance.

**FIG. 5.**
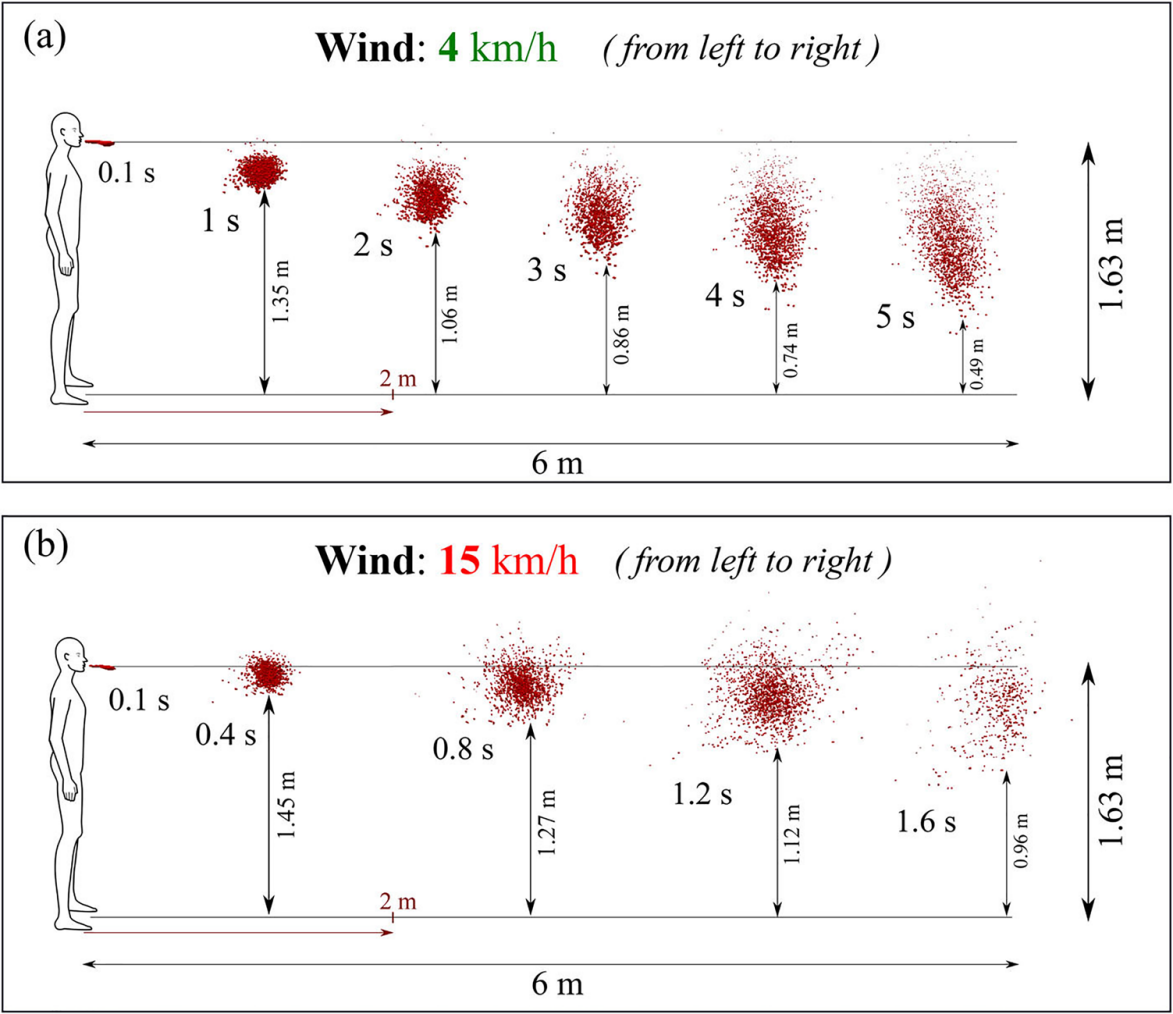
The effect of wind speeds of (a) 4 km/h and (b) 15 km/h on the transmission of saliva droplets. The wind is blowing from the left to the right, and the temperature and relative humidity are 20 °C and 50%, respectively. Reproduced with permission from T. Dbouk and D. Drikakis, “On coughing and airborne droplet transmission to humans,” Phys. Fluids **32**(5), 053310 (2020).^27^ Copyright 2020 AIP Publishing.

**FIG. 6.**
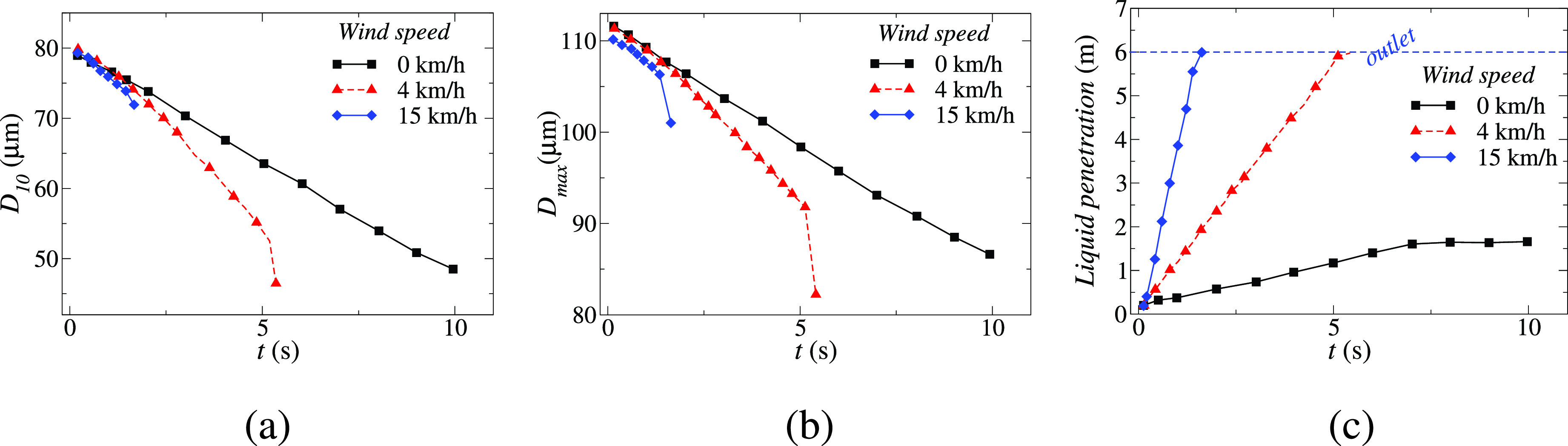
Temporal variation of the (a) mean saliva droplet diameter *D*_10_, (b) maximum saliva droplet diameter *D_max_* and (c) liquid penetration distance, which is defined as the maximum distance a saliva liquid droplet with a 95% initial mass can travel. Reproduced with permission from T. Dbouk and D. Drikakis, “On coughing and airborne droplet transmission to humans,” Phys. Fluids **32**(5), 053310 (2020).^27^ Copyright 2020 AIP Publishing.

Figure 5 shows the effect of wind speed on the transmission of saliva droplets. The dispersion, transport, and evaporation of the droplet ejected during cough are studied using computational fluid dynamics and heat transfer under different wind conditions.^27^ Saliva droplets normally fall to the ground at zero wind speed and do not travel more than one meter horizontally. Saliva liquid droplets can reach up to 6 m from the mouth in 5 s at 4 km/h wind speed blowing from left to right in the direction of the human cough, as shown in Fig. 5(a). This droplet cough cloud loses mass steadily over its traverse time through evaporation and wind shear-driven droplet breakup effects, and at 5 s the droplet cloud has lost over 60% of its initial ejected liquid mass. The saliva droplets fly in the form of clouds sheared by the wind and turbulent dispersion forces which cause significant cloud deformation. Some droplets fall on the ground while the entire cloud takes on an elongated elliptical shape that stretches from the throat level of the cougher to about 0.5 m above the ground by 5 s traverse time. When the wind speed is increased to 15 m/s under the same environmental conditions, as shown in Fig. 5(b), the saliva droplet cloud moves faster with an accelerated dispersion rate and reach 6 m in 1.6 s with an increased reduction in mass due to evaporation and wind shear induced enhanced droplet breakup effect. For concern, the droplet cloud remains approximately between the head and chest level of the cougher throughout which may enhance the chances of transmission. Figure 6(a) represents the variation in the mean saliva droplet diameter, *D*_10_, with time for various wind speeds. In all cases, the *D*_10_ diameter falls with time as the droplets lose mass through evaporation and breakup. However, the rate of decrease in mass increases as the wind speed is increased from 0 to 4 to 15 km/h as convection-driven evaporation and enhanced shear-driven droplet breakup processes are enhanced at higher wind speeds. Similar trends are seen for the largest droplets in the population which may constitute a greater risk of airborne disease transmission as they potentially carry larger viral loads. The variation of the maximum diameter of saliva droplets is plotted in Fig. 6(b). Reduction in maximum diameter of the droplets is observed from 111 to 82 *μ*m and reduction rate becomes faster with an increase in wind speed and a high shear rate of wind. However, since the droplet cloud moves faster at higher wind speeds, the enhances mass-loss rates are more than compensated by the faster traversal rates at high wind speed. Thus with high wind speeds, one is exposed to greater liquid masses at a given distance downwind of the cougher compared to lower wind speeds. This can be quantified by the liquid penetration distance, plotted in Fig. 6(c), calculated as the distance up to which 95% of the ejected liquid mass could travel. The liquid penetration distance was found to increase significantly with increased wind speed. With a 0 km/h wind speed, the liquid penetrations distance was below 2 m. However, the liquid penetration distance reached 6 m in 5.4 s for a wind speed of 4 km/h, while for a 15 km/h wind speed the liquid penetration distance attained a 6 m value in merely 1.6 s. This demonstrates the significant chances of transmission beyond the mandated 2 m social distance under downwind conditions.

A similar study shows, under a wind speed of 2 m/s, the 100 *μ*m droplet can fly up to 6.6 m.^32^ Dry environment conditions can further increase this distance. Small droplets having a size less the 50 *μ*m remain in air for a longer period and their dispersion distance is insensitive to relative humidity at the same temperature. The travel distance and lifetime of the large droplets may increase due to the reduction of volume in the evaporation. When two people are separated by one meter and have a high viral load, the cougher deposits more than 65% of the droplet volume on the listener. The amount of droplets that landed on the listener's body decreased considerably as the social distance was increased to 2 m. The droplet volume deposited on the listener ranged from 2 to 150 *μ*m in 90% of the cases. Based on the standard downward cough pattern, young children could be at greater risk than adults. Teenagers and short adults should keep a social distance of at least 2 m from taller people. According to Feng *et al.,*^33^ 2 m social distance is insufficient to prevent transmission of SARS-CoV-2 because of the complexity of environmental wind conditions. They have conducted a numerical study for wind velocities from 0 to 16 m/s with initial particle size from 2 to 2000 *μ*m under 40% and 99.5% relative humidity (RH) conditions. Two humans (the cougher and the recipient) were also simulated to be standing at a distance of 1.63 m (6 ft) from each other. The study found the presence of strong secondary recirculation zones in the wake region formed by the coughing person which traps the droplets within the wake. Thus any person within this wake region has a higher probability of exposure. The recirculation zones increase in intensity and flow complexity at higher wind speeds. Higher deposition fractions on both human bodies are caused by high relative humidity of 99.5%, especially at low wind speeds. Because high humidity amplifies the condensation effect, cough droplets continue to increase as they travel through the air until the partial pressure at the droplet surface meets the saturation pressure of water vapor. The impact of RH on the fraction of droplets deposited on the bodies decreases as wind speed increases, as the smaller droplets get more efficiently transported by the more intense circulation zones. Low RH percent causes the water in cough droplets to evaporate, resulting in a decrease in droplet size and they remain suspended in the air for a longer period. At wind velocity of 5 m/s or more, the volume fraction of liquid deposition on the healthy human did not decrease significantly even at higher separation distances of 3.05 m due to the effective transport of small droplets by the wind flow. Further, it was found that intermittent wind gusts are more efficient in droplet transport and deposition than steady-state wind flows. This points to the need for further studying the effect of intermittency in wind flow patterns on the transmission of saliva droplet clouds.

**FIG. 7.**
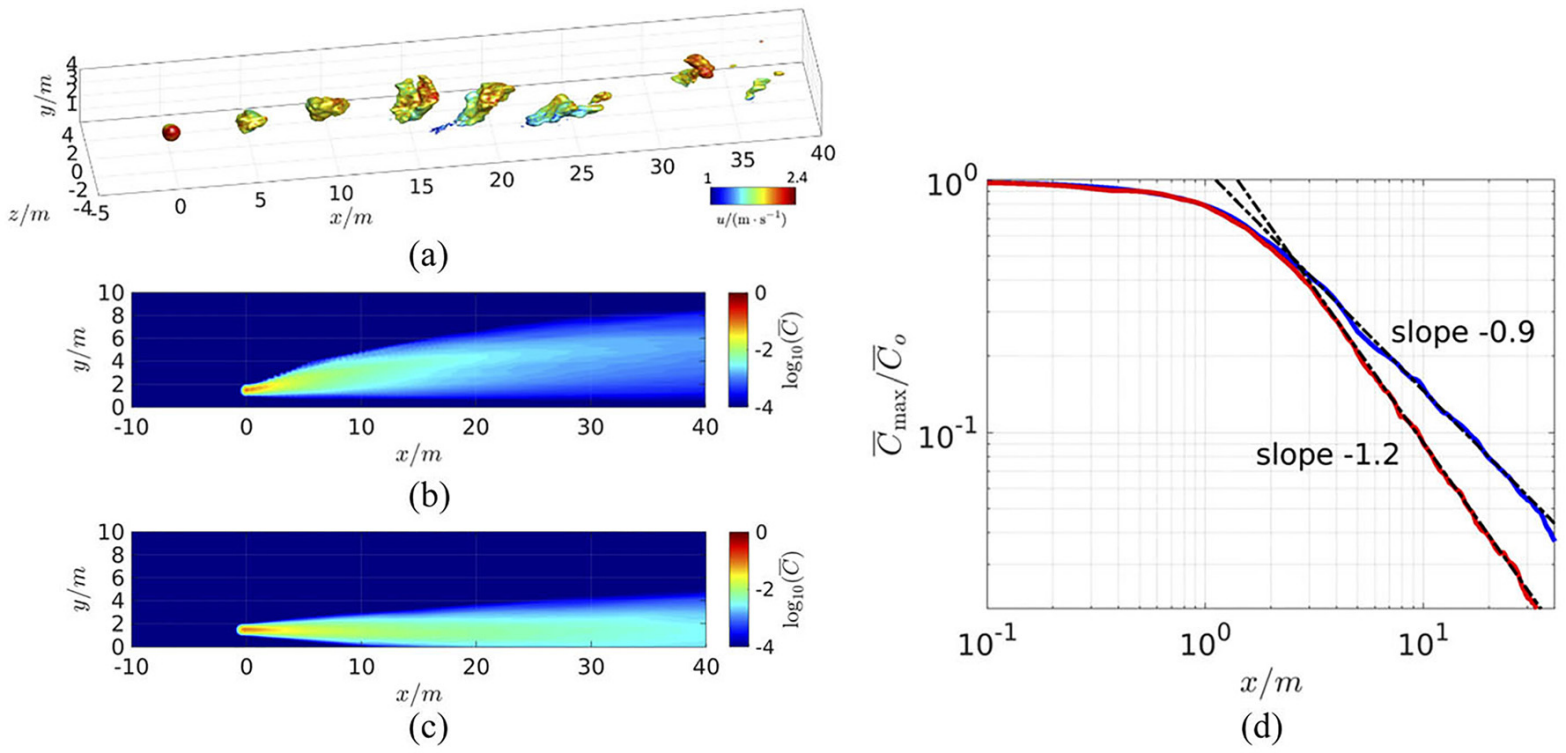
Numerical simulations of a breath-generated aerosol plume in a turbulent boundary layer using a large eddy simulation (LES). (a) The transit of breath aerosol puffs is represented by contours of $C/C_{o}=0.01$, (b) Mean concentration contours for a light plume (warmer than ambient temperature of $0{\;}^{{}^{\circ}}$C), (c) heavier plume (colder than ambient temperature of $42{\;}^{{}^{\circ}}$C), and (d) the mean concentration along the streamwise direction at 1.5 m above ground (in meters). The best-fit power laws beyond the near-field region are also shown. The temperature of the exhaled breath was assumed to be $37{\;}^{{}^{\circ}}$C. Reproduced with permission from R. Mittal, C. Meneveau, and W. Wu, “A mathematical framework for estimating risk of airborne transmission of COVID-19 with application to face mask use and social distancing,” Phys. Fluids **32**(10), 101903 (2020).^34^ Copyright 2020 AIP Publishing.

To study the effect of ambient temperature on the airborne transmission and the physical distancing between host and susceptible, the contagion airborne transmission (CAT) model is used.^34^ The CAT inequality is a mathematical model and belongs to the models used in epidemiology to predict the infection rates. CAT inequality considers three sets of variables depending on the host, environment, and susceptibility. To account for the effects like buoyancy and the time-dependent pulsatile nature of breathing in the present model, the data are used from a wall-modeled large eddy simulation. Here, the plume from a point source is located in a turbulent atmospheric boundary layer at 1.5 m above the ground with a 2 m/s average wind speed. The model is intended to simulate normal breathing by releasing the scalar (representing the respiratory aerosol) in 3 s intervals. The Boussinesq approximation is used to include the buoyancy effect. Two atmospheric temperature conditions are considered: $0{\;}^{{}^{\circ}}$C and $42{\;}^{{}^{\circ}}$C, and the temperature of exhaled breath is assumed to be $37{\;}^{{}^{\circ}}$C. Figure 7(a) presents the instantaneous scalar concentration at $42{\;}^{{}^{\circ}}$C ambient temperature. Figures 7(b) and 7(c) predict the time-averaged plume concentrations at $0{\;}^{{}^{\circ}}$C and $42{\;}^{{}^{\circ}}$C, respectively. In the first case, the plume is hotter than ambient air and rises (light plume), whereas in the latter case surrounding air is hot and the plume moves downward (heavy plume) due to the buoyancy of the air. Beyond the distance of approximately 3 m, the plume concentration decays consistently as $C(x)∼(x^{-1.2})$ and $C(x)∼x^{-0.9}$ for the light and heavy plumes, respectively, as shown in Fig. 7(d). This result shows that the maximum concentration of the respiratory plumes as a function of distance in both the scenarios. Here, $C(x)=C_{\max}(x)/C_{0}$ is the normalized maximum concentration at any given distance from the host at a height of 1.5 m above ground and x is the distance from the point source. *C*_0_ is the aerosol concentration near the host and *C_max_* represents the maximum concentration of the respiratory plumes. It can be concluded the concentration decay rate is affected significantly by the buoyancy and presence of ground.

**FIG. 8.**
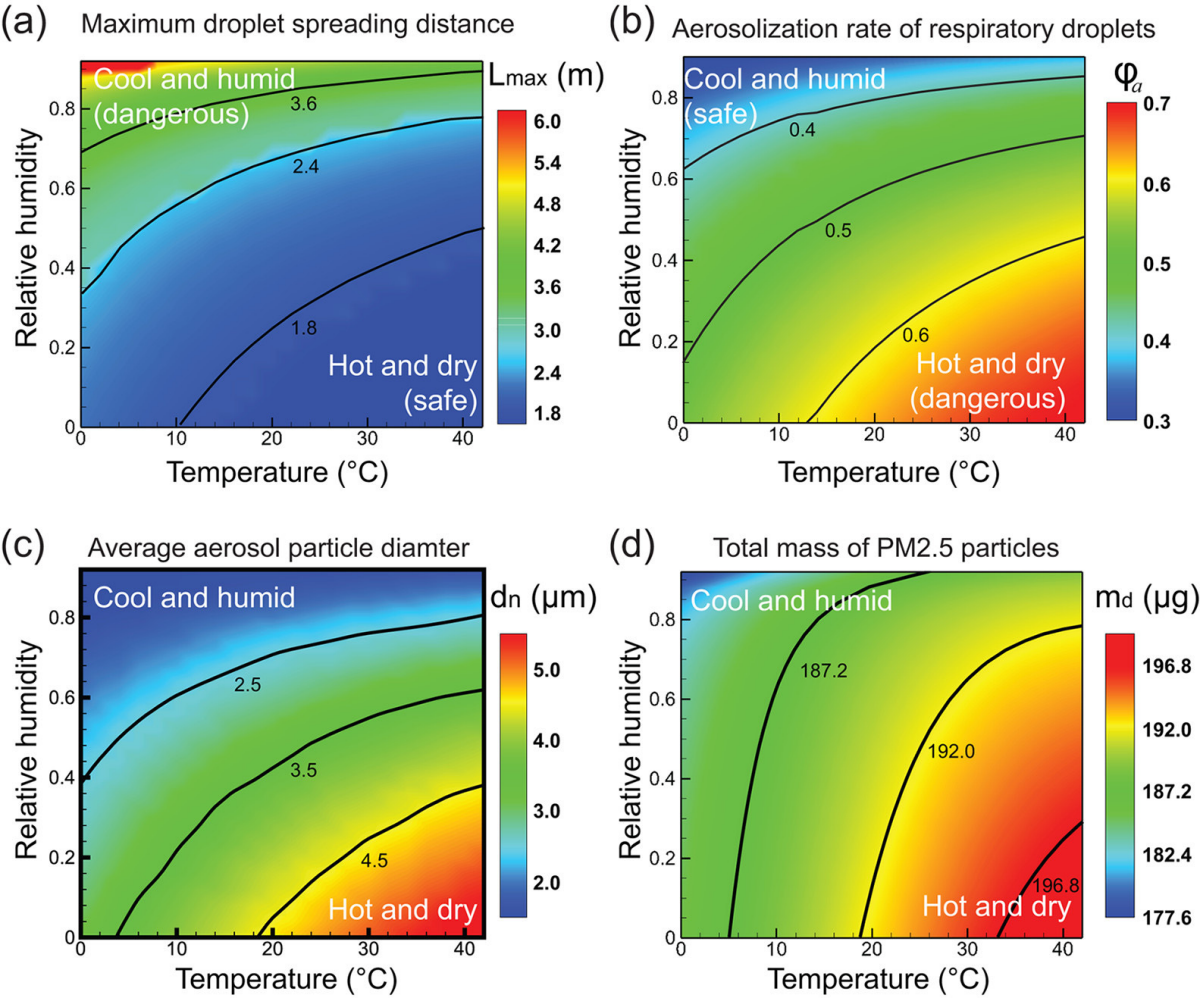
The effect of ambient temperature and relative humidity on (a) maximum droplet spreading distance, (b) respiratory droplet aerosolization rate, (c) average aerosol particle diameter, and (d) total PM2.5 particle mass. Here, ambient temperature is varied from $0{\;}^{{}^{\circ}}$C $-42{\;}^{{}^{\circ}}$C and relative humidity is varied from 0−0.92. Reproduced with permission from Zhao *et al.*, “COVID-19: Effects of environmental conditions on the propagation of respiratory droplets,” Nano Lett. 20 (10), 7744–7750 (2020).^35^ Copyright 2020 American Chemical Society.

The effect of ambient conditions on the transmission of speech respiratory droplets is explored, and particle motion, accumulation, and deposition of respiratory particles are described using kinematic equations.^35^ The ambient temperature range under consideration is 0–42 °C and relative humidity ranges from 0 to 0.92. The maximum horizontal distance traveled by all respiratory droplets, *L_max_* is plotted under different RH and temperature conditions as shown in Fig. 8(a). Under a hot and dry environment, respiratory droplets evaporate faster and decrease in size due to the growing damping effect of air. In contrast, the droplets travel a longer distance and reach as far as 6 m in a cool and humid environment. In most regions, droplets travel a distance of more than 1.8 m. This implies the current social distancing limit of 6 ft may be insufficient to prevent the transmission. Next, the aerosolization rate which is the percentage of droplets that become aerosol particles ϕ is evaluated under different weather conditions. Figure 8(b) predicts an increasing aerosolization rate for hot and dry settings, in contrast to the pattern observed for *L_max_*. Based on the results, the terminal size of the aerosol particles is found to be in the range of 1–15 *μ*m. These small size aerosols can accumulate in public places like hotels, hospitals, offices and schools due to their potential to suspend in the air for hours before settling on the ground. As a result, the long-range transmission of aerosol particles demands additional attention in the summer, particularly in dry weather. To prevent aerosol transmission, wearing face masks is one of the important preventive measures. The filtering efficiency of the face masks depends on the size of the particulates. Under various environmental conditions, the average diameter of the aerosol particle is computed. After dehydration, the final size of an aerosol particle is calculated by estimating the volume of sodium chloride dissolved in the aqueous solution. Only salt is taken into account when estimating the size of aerosol particles because the diameter of the virus is negligible when compared to salt in a respiratory liquid.^36^ Figure 8(c) shows the maximum droplets are less than 10 *μ*m in diameter with average diameters between 2 and 5 *μ*m, which have high potential to enter the human respiratory system. Due to the enhanced evaporation rate in dry and hot weather, the average particle size is increased as more respiratory droplets are converted to aerosols particles. Figure 8(d) demonstrates the total mass of particulate matter 2.5 (PM2.5) under different conditions as they have a higher potential of entering the lung. Due to their smaller size, they remain suspended in the air for a longer time and size-dependent suspension time $\left(t_{s}\right)$ is calculated using the equation

$$v_{e}t_{s}+(v_{t}-v_{e})\tau \left(1-\text{exp}\left(\frac{-t_{s}}{\tau }\right)\right)=\frac{L_{m}}{2}-L_{z},$$ (2)

where *v_e_* denotes the aerosol particle's falling velocity assuming that buoyancy and air drag completely balance gravity, *v_t_* denotes the aerosol particle's downward velocity at the time of evaporation, and *L_z_* represents the vertical displacement. The time constant *τ* for microsized particles is given as (neglecting Brownian motion)

$$\tau =\frac{2\rho_{d}r^{2}}{9u_{a}},$$ (3)

where *r* denotes the radius of the droplet, *ρ_d_* is the droplet density, and *u_a_* is the dynamic viscosity of air. The time constant *τ* is less than 0.05 s for the droplets less than 100 *μ*m which indicates the strong damping effect of air. As shown in Fig. 8(d), PM2.5 is less in the cold and humid environment than in the hot and dry environment due to the increased suspension time of the particles. From Figs. 8(b) and 8(d), we can conclude that the percentage of droplets turning to aerosols is more and PM2.5 particles are buildup in confined space under hot and dry weather conditions. Improper airflow will significantly increase the moving distance of droplets and aerosol particles, increasing the risk of COVID-19 transmission. As a precautionary measure, a healthy person should follow different social distancing different in still and flowing air environments.^8^ In some counter-examples, such as in India, the prediction that high humidity and high temperatures would minimize the incidence of coronavirus cases was not found to be consistent.^37^

### B. Face mask and social distancing

**FIG. 9.**
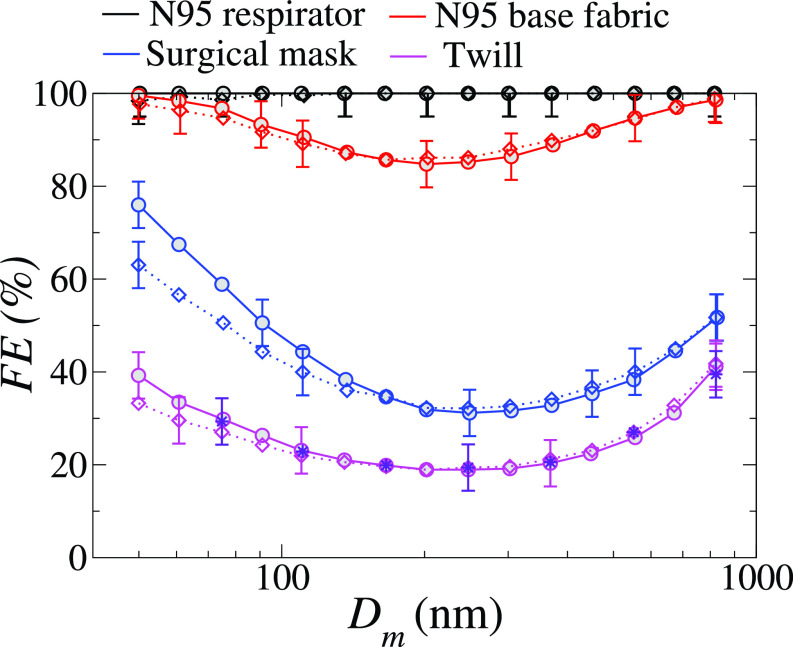
Filtration efficiency (*FE*) of an N95 respirator (black), the N95 base fabric (red), a surgical mask (blue), and a twill (magenta) as a function of particle mobility diameter (*D_m_*). The basic sample is represented by solid bold lines, whereas the reneutralized samples are represented by dashing lines. The indigo starts to represent the twill FE as determined by an aerosol particle mass analyzer and reneutralization. In FE, the uncertainty is 5%. Reproduced with permission from Zangmeister *et al.*, “Filtration efficiencies of nanoscale aerosol by cloth mask materials used to slow the spread of SARS-CoV-2,” ACS Nano **14**(7), 9188–9200 (2020).^39^ Copyright 2020 American Chemical Society.

Daily observations show a plethora of medical and social conditions in which maintaining social distancing is either unfeasible or not practiced properly during the COVID-19 pandemic.^40^ The previous discussion has also highlighted the insufficiency of the 6 ft social distancing rule in protecting the individual from being exposed to potentially viral-laden respiratory droplets under various plausible meteorological conditions. One of the widely accepted preventive measures is the wearing of a face mask. The effectiveness of the face mask is measured in terms of filtration efficiency. Filtration efficiency is defined as the percentage of the contaminant (here the liquid droplets in the exhaled air jet) by the mask filter. Thirty-two cloth materials are used in the making of various types of masks^39^ to study the filtration efficiency (*FE*), quality factor (*QF*), differential pressure (ΔP), and construction parameters. Here, the quality factor is calculated as $QF=-ln(1-FE_{\min}/100)/\Delta P$. The size of the SARS-CoV-2 virus under consideration was 100 ± 10 nm. Figure 9 shows the filtration efficiency of the most commonly used mask for different particle mobility diameters (*D_m_*) of the particles. The filtration efficiency is calculated using the expression

$$FE=1-\text{exp}\left[\frac{-4E_{f}\alpha L}{\pi D_{f}}\right],$$ (4)

where *α*, *L*, and *D_f_* represent the material porosity, filter thickness, and fiber or yarn diameter, respectively. The single-fiber efficiency *E_f_* is the sum of impaction (EI), diffusion (ED), interception (ER), and electrostatic deposition (EB). Here, impaction is the process through which big particles collide or impact with the fiber inertially. The droplet-fiber interaction is dominated by molecular diffusion and Brownian forces for small particles; for large droplets, the fiber particles can actively intercept a droplet when the distance between them is less than one particle radius; and for some materials, the droplets may be deposited due to electrostatic charge differences between the fiber and the droplet. After size selection, the aerosol had a moderate net charge (*q*, where 1≤q≤4, approximately) due to charge neutralization before measurement. The effect of particle charge on FE was investigated by measuring the FE of seven samples that had been neutralized following size selection and represented by dashed lines in Fig. 9. The measured data shows that particle charge has only a minimal effect on the FE of the textile materials studied. Furthermore, the FE of twill (polyester/cotton blend 3) was determined by passing the selected aerosol size via an aerosol particle mass analyzer (APM) and then neutralizing it. Aerosol neutralization (and mass selection) had no effect on FE except the surgical mask, which uses polypropylene layers for filtration. Using these data, Mittal *et al.*^34^ calculated the upper and lower bound of the average FE $\left(\bar{FE}\right)$ for few fabric samples for the particle size range from 50 to 5 *μ*m. The cotton 4, cotton 14, and synthetic blend 2 provide a similar or better protection factor than the surgical mask. It can be concluded that face masks made from all these fabrics could considerably reduce the overall transmission rate.

Akhtar *et al.*^40^ also investigated the effectiveness of face masks made up of different materials. Experiments are performed for N-95, surgical, cloth, cloth PM 2.5, and wetted cloth PM 2.5 type masks. Two configurations are considered: (1) a healthy person wearing a mask for protection and (2) an infected person wearing a mask to prevent the spreading of the virus using particle image velocimetry. The leakage percentage of the airborne droplets is expressed in terms of the number of virus particles. The N-95 mask proved to be the most effective and showed zero leakage of airborne droplets. Thus N-95 masks could protect the wearer from transmission even for face-to-face interactions. However, it is observed that most types of face masks could not save the healthy person without social distancing for moderate to high viral loading. The mask with two layers of dry fabric is the least effective. Due to enhanced exhale and inhalation rates of the individuals participating, the transfer risk in a facility such as a gym, where exercise intensity levels may be intense, the transmission risk might be over 200 times higher.^34^ A comparison is made for medical grade and nonmedical grade face mask for indoor (stagnant airflow) and outdoor conditions (mild unidirectional breeze) for the particle size from 10 to 300 *μ*m.^41^ Saliva droplets could disperse up to 2.62, 0.73, and 0.48 m during indoor coughing in cases without the mask, nonmedical and medical-grade grad mask, respectively. The distance traveled by the 10 *μ*m particles due to saliva evaporation is increased to 2.84 m and 0.91 m without the mask and in the presence of a nonmedical grade mask, respectively. Few experimental studies are evaluated by Wei *et al.*^42^ for various types of masks and filter materials considering the different range of particle size. All types of face masks can block large droplet particles and can filter out the vast majority of the viruses. Filtration efficiency for large and extra-large particles is close to 100% for all kinds of masks.

**FIG. 10.**
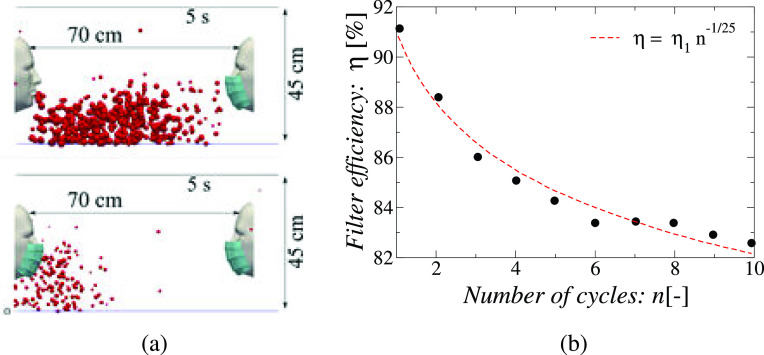
(a) People who use masks reduce respiratory droplet transmission while (partially) isolating themselves from coughing. The findings of a 5 s simulation time for a surgical mask with a 91% initial efficiency are provided. The temperature of the skin of the face is 32 °C, whereas the temperature of the mouth is 34 °C. In this simulation, there is no wind, the ambient temperature is 20 °C, the pressure is 1 atmosphere, and the relative humidity is 50%, (b) Variation of effective dynamic filter efficiency (*η*) with the number of cycles *n* of a coughing event, $\eta_{1}=90.4\%$. Reproduced with permission from T. Dbouk and D. Drikakis, “On respiratory droplets and face masks,” Phys. Fluids **32**(6), 063303 (2020),^43^ Copyright 2020 AIP Publishing.

Dbouk and Drikakis^43^ investigated the droplet dynamics caused by a mild repeated coughing incident and the fluid dynamics phenomenon affecting mask performance using multiphase computational fluid dynamics considering evaporation, droplet phase-change, turbulent dispersion forces, droplet phase-change, and breakup. The researchers simulated ten cough cycles over a total period of 5 s under quiescent air conditions. Each cough caused droplet-laden air jet to be ejected at the speed of 5 m/s over a period of 0.12 s after which the mouth closes. It is found that repeated coughing events increase the number, the residence time and the distance traveled by the droplets as jets emitted in the later cycles push the droplets from previous cycles further downstream and the droplets and multiple ejected puffs interact and delay the dispersion of the droplets in the ambient air. However, the use of a mask with an initially 91% FE reduces the number of droplets that escape into the ambient. The distance traversed is also reduced, it is found that the droplets travel 70 cm without a mask, and this distance is reduced to half after wearing the mask as shown in Fig. 10(a). The liquid penetration distance also falls from 42 to 22.38 cm when a mask is worn. The Sauter mean diameter of the droplets ejected during coughing decreases from 75 *μ*m to 55–50 *μ*m when the mask is worn. Importantly, the filter efficiency (FE or *η*) of the mask falls as the number of coughs increase. For the ten cycle cough simulated by the authors, the FE decreased from the rated 91% to 82%, and the fitting function of $\eta =\eta_{1}n^{-0.04}$ was found to match the results well, where *η*_1_ is the rated FE,*η* actual FE and *n* is the number of cough events [see Fig. 10(b)]. This indicates that day-long usage with multiple cough events may significantly reduce the effectiveness of a mask that has a high-rated filtration efficiency. The mask-to-face fitting is an important factor that affects airborne transmission because even a small opening can lead to extra leakage of droplets around the mask. The generation of secondary flows leakage through the gaps left at the mask's edges due to its imperfect fit should also be considered while evaluating the performance of the face mask not only their particle filtering capacity.^44^

Many studies suggested the use of a proper face mask to prevent the spread of COVID-19 without considering CO_2_ exhaled during the respiratory process.^45^ Excess inhalation of CO_2_ (hypercapnia) leads to muscular weakness, discomfort, fatigue, headaches as well as drowsiness. A suitable face mask that helps to exhale the released CO_2_ into the atmosphere should be recommended. A full mask respirator is suggested due to no snorkel clearing, less fogging due to better air circulation, no jaw fatigue, and suitable for colder climates. The use of a face mask in a windy region should be imposed even the person is not in public as the droplets are transported by the wind. Although mandating a face mask is not enough to prevent a COVID-19 resurgence, it can help to decrease the infections.^46^ In the classroom context, masks have been shown to benefit from interacting with the thermal plume produced by natural convection caused by body heat, which moves aerosols vertically away from neighboring students.^47^ A social distance of 1.6–3 m for the activities like breathing and talking is recommended, but this distance can be up to 8 m considering all the aerosols under a calm environment.^38^

### C. Indoor vs outdoor transmission

The fact that an asymptomatic patient triggered an air-conditioner-induced COVID-19 outbreak in a restaurant in Guangzhou, China^48^ alerts us to potential outbreaks related to ventilation in public places. Usually, people spend most of their time in transportation and residential buildings than outside.^38^ Therefore, ventilation systems play an important role in controlling the spread of COVID-19. Analysis shows that the infection is linearly dependent on the exposure time. The required ventilation rate is significantly reduced by increasing social distancing. For example, if the occupancy rate is reduced by 25% in an office, the ventilation rate can be reduced by four-fifth for the first 30 minutes of exposure and to 40% for the public bus. The standard-required minimum fresh airflow is not enough to reduce the infections. A ventilation rate of 1 l/s per person is enough for the airborne transmission of the SARS-CoV-2 in crowded space.^49^

**FIG. 11.**
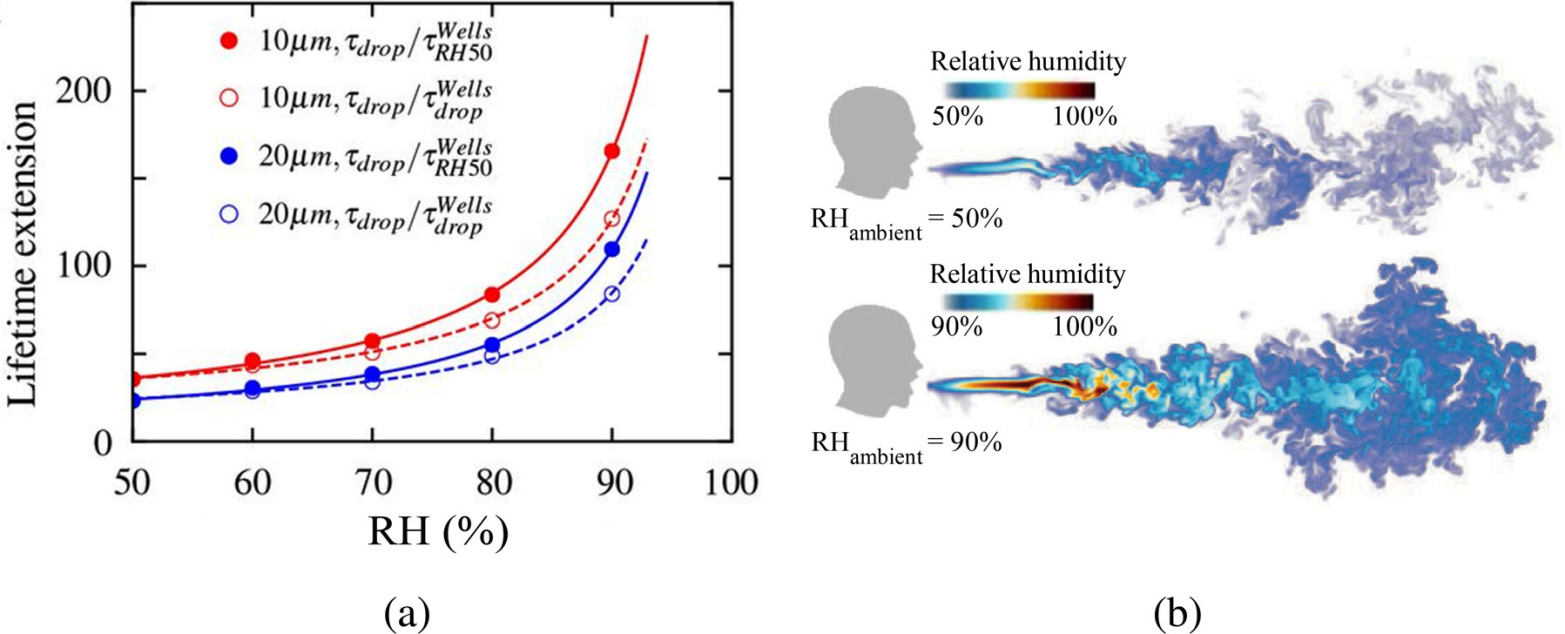
(a) Variation of the lifetime extension by number of factors as compared to the lifetime of a droplet behaving according to the Wells model^51^ with the relative humidity. (b) Relative humidity field at 600 ms when the ambient RH is 50% and 90%. Reproduced with permission from Chong *et al.*, “Extended lifetime of respiratory droplets in a turbulent vapor puff and its implications on airborne disease transmission,” Phys. Rev. Lett. **126**, 034502 (2021).^50^ Copyright 2021 American Physical Society.

The evolution of a droplet-laden puff was explored by direct numerical simulations of a turbulent coughing event in order to understand the safety demands in an indoor environment.^50^ Over a 0.6 s time span, the Lagrangian statistics of the released droplets were tracked. A jet of saturated air (100% RH) containing 5000 water droplets was discharged in an ambient of $24{\;}^{{}^{\circ}}$C under quiescent conditions typical of residential environments to simulate a cough. The ambient relative humidity values were taken to be between 50% and 100%. One of the study's significant findings was that the population's lifetime was far longer than previously assumed value because the microscopic droplets became trapped in the humid puff of ejected air throughout their travel. As the ambient humidity level increased, the prolonged lifetime becomes more prominent. At RH = 100% and for smaller droplets, the lifetime of the tiny droplets grows substantially as seen in Fig. 11(a). Specifically, the lifetime of the smallest respiratory droplets with initial diameter *d_p_* = 10 *μ*m, RH = 90% is extended by a factor of about 130. This is in line with the classical Wells model,^51^ which projected a factor of 166 for RH = 50% using the isolated droplet assumption. Similarly, somewhat bigger droplets with an initial diameter of *d_p_* = 20 *μ*m exhibit a considerable increase in lifetime of 80–110 times. The reason for the considerable increase in droplet lifetime shown in Fig. 11(a) is a significant decrease in the evaporation rate due to the higher puff humidity. The other reason is that when the ambient RH is higher, the vapor puff might stay longer, as seen in Fig. 11(b).

The presence of strong ambient mean flow and turbulence, whether from indoor ventilation or outside cross-flow, will have a significant impact on the virus-laden droplet dispersal.^11^ Due to long exposure times and low turbulence level (and thus dispersion), the indoor virus transmission far exceeds outdoor transmission.^13^ Building ventilation is needed to reverse this situation and to avoid secondary outbreaks. Different ventilation systems are studied for indoor environments such as mixing ventilation, displacement ventilation (natural and mechanical), and wind-driven ventilation. Displacement ventilation is found to be the most effective. Displacement ventilation (either mechanical or natural), where extractions are at a high level and intakes are at a low level, creates negative pressure at the occupant level, which pulls in fresh air from outside, and positive pressure near the ceiling, which pushes hot, dirty air out. Displacement ventilation is recommended for public places (supermarkets, bars, restaurants, etc.) to minimize the further spread of disease. In a natural ventilation system, the hot buoyant air released due to body heat and equipment inside rooms travels upward toward the ceiling and exits through the opening provided in the room. This air carries aerosols with it and flushed them out of the building. The cooler outdoor air enters the room which flows across the floor. The plumes are emitted at varying heights from the several heat sources in the room, and the volume of the room below the lowest heat source is unimportant because it contains air at the ambient outdoor temperature. The interface separates the cool, unpolluted zone below from the heated, contaminated zone above at a height of *h*. The room's effective height is *H* − *hv*, where *H* is the floor-to-ceiling height and *hv* is the lowest plume's virtual origin. The interface height is unaffected by the strength of the heat sources and is only specified by the quantity of open space in the case of *n* inhabitants, represented by equal strength plumes with the same virtual origin heights. This open area is calculated as

$$A=nC^{3/2}\frac{h^{5/2}}{\sqrt{H-hv-h}},$$ (5)

where *C* is an empirical constant with an approximate value of 0.105 and *n* denotes the number of inhabitants. The open area *A* depends upon the top opening area (*a_t_*) and bottom opening area (*a_b_*) and is given by the expression

$$A\approx \frac{ca_{t}a_{b}}{\sqrt{\frac{1}{2}(a_{t}^{2}+a_{b}^{2})}}.$$ (6)

The discharge coefficient c≈0.6. Natural displacement ventilation works best in buildings with high ceilings and broad openings.

Due to the lack of upper-level openings or open space in the buildings, the natural ventilation may not be effective and can be replaced by the mechanical ventilation system. For n occupants in the room, the total extraction rate of warm air is given by the equation

$$Q=n^{2/3}CB^{1/3}{\left(h-hv\right)}^{5/3},$$ (7)

where *B* is the buoyancy force produced by *n* sources. In theory, given an appropriate mechanical ventilation rate, *h* − *hv* can be set to any height.

**FIG. 12.**
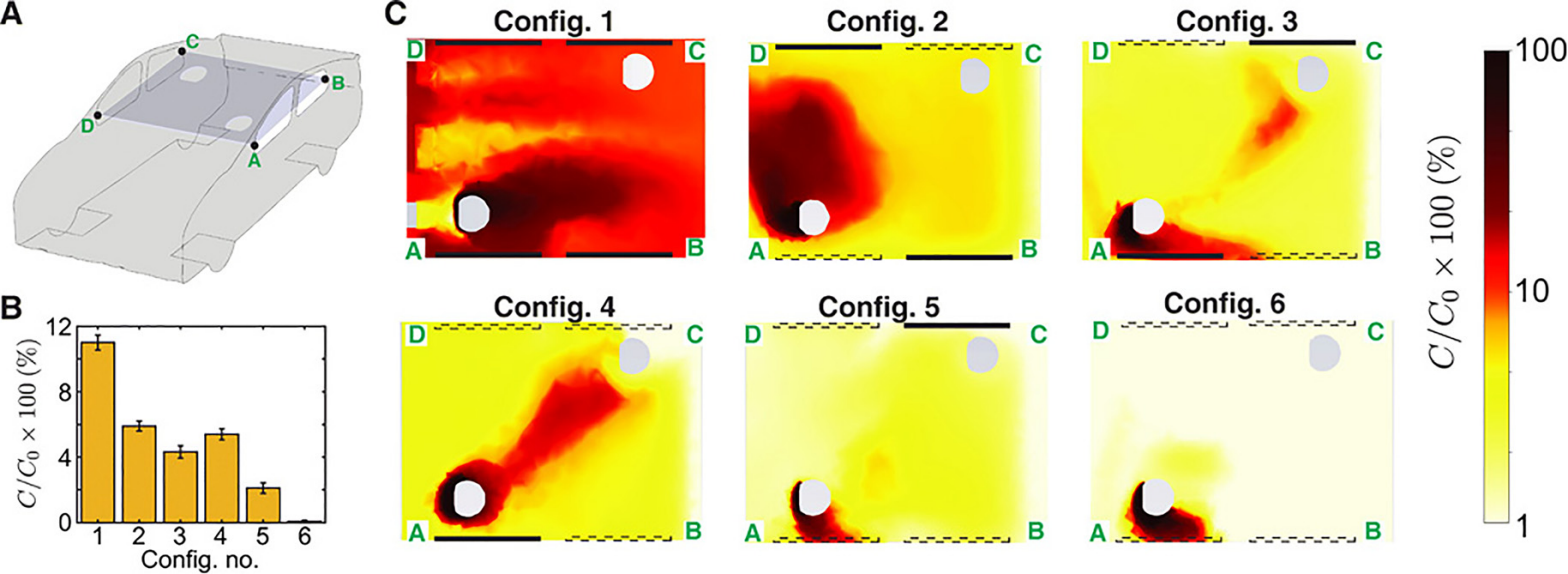
(a) Concentration fields are displayed on a schematic of the car with a cut plane passing through the center of the interior compartment. (b) The bar graph illustrates the mass fraction of air that reaches the passenger from the driver. Standard deviation of the concentration field around the passenger is represented by the error bars. (c) The heatmaps displaying the concentration field of the species originating from the driver for various window situations. The line segment A–D is at the front of the car cabin, and the flow direction in panel C is from left to right. Open windows are represented by dashed lines, whereas closed windows are represented by solid lines. *C*_0_ is the initial mass percentage of passive scalar at the injection site, with $C/C_{0}$ = 1. Reproduced with permission from Mathai *et al.*, “Airflows inside passenger cars and implications for airborne disease transmission,” Sci. Adv. **7**(1), eabe0166 (2021).^55^ Copyright 2021 American Association for the Advancement of Science.

A numerical study is conducted for aerosol transmission in the classroom.^52^ Particles greater than 50 *μ*m were found on the desk, the ground, and other surrounding surfaces in the room. The transmission of 1 *μ*m particles from a source individual to others separated by at least 2.4 m is decreased by 92% when glass barriers are used. When windows are open, the particle escape percentage increases by 38% compared to closed windows, and 69% of the particles leave the system. When the windows are open, the amount of aerosol that collects on the students is reduced. The need for efficient filtration and sterilizing systems inside air conditioners is highlighted by the fact that a large fraction of inhaled particles ends up in the air conditioning system. In the context of a classroom scenario, ventilation with moderate filtration has been shown to substantially reduce the likelihood of infection transmission.^47^ The probability of aerosol transmission is high in the indoor environment where physical activity is intensive, like gym due to deep inhalation and exhalation of air.^53^ To reduce the aerosol particle concentration inside the gym, it is suggested that a costly redesign to the current mechanical ventilation system be avoided. It could instead be accomplished by supplementing this system with mobile specialist high-quality AC systems. Mounting AC systems near ground level in gyms with a high ceiling (e.g., 5 m) can be more efficient than the ventilation system. In public transport like buses, aerosol transmission is studied for particles ejected from coughing and sneezing for turbulent and laminar flow of the droplets with the help of trackers.^54^ It is found that, when the bus speed exceeds 40 km/h, the particles are unable to advance farther because of high disturbances generated by air via the windows, which forces the particles backward, affecting only the passengers sitting or standing behind the afflicted passenger. In contrast, the propagation is stronger when the bus is at rest or stops in a bus station, reaching a wider distance and having a greater impact on the passengers in that region. As a result, it is preferable to minimize halts and provide a limited number of bus stations. Matahi *et al.*^55^ have performed a RANS-based simulation of aerosol transmission inside a left-hand-drive car to estimate the probability of exposure of either the passenger or the driver to COVID-19 containing aerosol droplets ejected by the other. The passenger is seated diagonally with respect to the driver at a distance of 1.5 m and the car is traveling at 50 miles/h. Several configurations are analyzed, from the case where all windows are closed with the car air-conditioner operating at the “fresh” setting, to the case where all windows are open. It was found that the rate of transmission is higher, in general, when the driver is the source and the passenger is the recipient of the virus-containing aerosols than vice versa. This case has been shown in Fig. 12. Closed windows have been shown as thick black rectangles and the open windows are shown with dotted rectangles. The rate of aerosol transmission from the driver to the recipient is highest, near 11% for the case where all the windows are closed and only the car air-conditioner is running. But the transmission rate drops to less than 1% when all the windows are open. Interestingly, among the intermediate configurations, the largest transmission of 5% occurs for the case where the windows adjacent to both the driver and the passenger are open. This counterintuitive result can be explained by the fact that a flow of air develops between the driver-side window which acts as an inlet and the passenger-side window that acts as an outlet due to the pressure differential between the windward and leeward sides of the moving vehicle. The study shows that only by opening all the windows can the transmission probability be reduced to safe limits within a car. In a crowded confined area like an elevator, the study of airborne transmission is important.^56^ The numerical study is conducted for turbulence flow of droplets ejected during coughing. The droplets generated during coughing fall on the ground when the fan is switched on. Cough droplets can circulate in the elevator if the fan is turned off. This is due to the flow field created by the air puff and cough droplets expelled together. It is recommended that the elevator fan be turned on, and a centrally positioned fan is preferred overspread air supply. Particle transports in the indoor air show complex dynamics and no linear dependence on air velocity.^57^ Combining face masks with either the WHO or the CDC social distancing guidelines should be quite helpful in reducing infection in an indoor environment.^41^

## III. SURFACEBORNE TRANSMISSION

During coughing, sneezing, or talking, droplets with diameter larger than 10 *μ*m land on the ground/surface under gravity. Along with the virus, these droplets contain several other ingredients, such as salt (NaCl), protein (mucin), and surfactant (dipalmitoylphosphatidylcholine).^4^ The lifetime of virus-bearing droplets on different surfaces determines surface contamination, which is further influenced by several factors, such as ambient temperature, relative humidity, composition, droplet volume, contact angle, etc. The equilibrium contact angle (*θ*) of a sessile droplet on various surfaces, as well as the ambient temperature (*T*) and relative humidity (RH in %) encountered in various locations around the world, is summarized in Table II. Sections III A–III F examine the evaporation dynamics of respiratory sessile droplets on surfaces and reviews the research on how environmental conditions affect the lifetime of these droplets.

**TABLE II.** Typical values of the contact angle (*θ*) of a sessile droplet on different surfaces and the ambient temperature (*T*) and relative humidity (%) experienced in different cities around the world (taken from^58–63^ and https://weather.com).

Surface	Representative contact angle (*θ* in degree)	City	Ambient temperature (*T*) (°C)	Relative humidity (RH) (%)
Glass	10	New York	(−3)–30	52–91
Stainless steel	30	London	4–20	46–85
Cotton	50	Mumbai	18–40	27–95
Wood	70	UAE	19–40	30–90
Smartphone	90	Delhi	9–33	42–90
		Indoor (AC)	22	30–50

### A. Methods

#### 1. Mathematical modeling

**FIG. 13.**
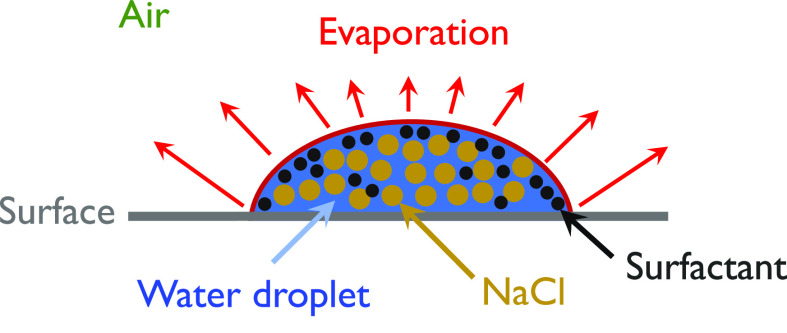
Schematic of a sessile saliva droplet on a surface.

The various phenomena affecting the evaporation of a normal sessile droplet are diffusion, free convection, and passive transport.^64–70^ The evaporation dynamics of a sessile droplet in the presence of surfactant and other ingredients is more complex due to the associated contact angle dynamics, and thermo-solutal Marangoni flow.^71,72^ However, in the case of a very small droplet [volume, V≤O(1) *μ*l], the dominance of surface tension over gravity force resulted in a symmetrical cap profile, and a diffusion-based model was found to adequately predict its lifetime. A few notable contributions to the prediction of the lifetime of respiratory droplets can be found in Refs. 63, 73, and 74. A computational model based on kinetic theory was used to investigate the drying time and time-varying thickness of a liquid film over solid surfaces.^73^ A diffusion-based theoretical model was used in Refs. 63 and 74, to estimate the drying time. Unlike, the pinned droplet with a constant contact angle as assumed by Bhardwaj and Agrawal,^63^ Balusamy *et al.*^74^ considered the dynamic contact angle of saliva droplets laden with salt and insoluble surfactants (see Fig. 13). The diffusion-based evaporation rate ($\dot{m}$ in kg/s) of a sessile droplet of wetting radius (*R*) is given by

$$\dot{m}=-\pi RD(T)C_{\mathrm{sat}}(T)\left(1-\text{RH}\right)f(A)\alpha ,$$ (8)

where $f(A)=1.3+0.27{\left(\frac{\theta (t)\cdot \pi }{180}\right)}^{2}$ for $\theta \leq 90^{{}^{\circ}}$. The value of *α* is a constant that ranges from 0.267 to 1. In Ref. 74, the molality (*M*) of the saliva is fixed at 0.154 mol/kg. D(T) represents the diffusion coefficient of water vapor ($m^{2}/s$), which is given by^63^

$$D(T)=2.5\times 10^{-4}\;\text{exp}\;\left(\frac{-684.15}{T+273.15}\right).$$ (9)

The saturation vapor density, *C_sat_* (in kg/m^3^), is calculated using Raoult's law for water-salt-surfactant mixtures,

$$C_{\mathrm{sat}}=X_{w}C_{\mathrm{sat}}^{{}^{\circ}},$$ (10)

wherein the saturation vapor density of pure water, $C_{\mathrm{sat}}^{{}^{\circ}}=9.99\times 10^{-7}T^{3}-6.94\times 10^{-5}T^{2}+3.20\times 10^{-3}T-2.87\times 10^{-2}$ and *X_w_* denotes the mole fraction of water in the solution. The accommodation coefficient *α* is obtained as^71^

$$\alpha =\frac{1}{1+\Psi \frac{\Gamma }{\Gamma_{\infty }}}.$$ (11)

Here, the surfactant parameter, Ψ≥0 and Γ and $\Gamma_{\infty }$ are the instantaneous concentration of the surfactant (obtained by calculating the moles of surfactant, solute ion particles and water in the droplet at any time, *t*) and its maximum value corresponding to the fully dried drop. Finally, the rate of change in the droplet volume (dV/dt) is obtained as $\dot{m}/\rho$ using the density of the solution *ρ* at a given time.^75^

#### 2. Experiments

Several researchers (e.g., Refs. 76 and 77) studied the effect of evaporation of organic solvents and saltwater droplets on surfaces, while a few others (e.g., Ref. 78) studied the evaporation of saliva droplets on surfaces in context with COVID-19. Lieber *et al.*^78^ used an acoustic levitator in conjunction with microscopic imaging and particle image velocimetry (PIV) to study the evaporation characteristics of saliva droplets under well-controlled ambient conditions. An acoustic levitator with a resonance frequency of 100 kHz was used to levitate a saliva droplet with a size ranging from 10 to 1000 *μ*m inside a controlled chamber (i.e., at fixed temperature and relative humidity). The saliva probes of two healthy men were taken from their mouths and inserted into the acoustic levitator using a syringe in order to study the evaporation dynamics. It is observed that the acoustic streaming causes an increase in evaporation rate that is comparable to forced convection at a relative velocity of around 0.1 m/s. Basu *et al.*^77^ studied the drying and precipitation characteristics of the saltwater droplets in a similar levitator using a shadowgraph technique. He *et al.*^79^ used an inverted microscope and a CMOS camera in their experiments and found that during the evaporation, a droplet of size ranging from 5 to 100 *μ*m shrinks to a few micrometers in size (referred to as residues). They found that the surfaces and humidity of the environment have a significant effect on residue formation. When relative humidity is less than 40%, they found that over 80% of droplets form residues on plastic and uncoated and coated glass, but only 20% form on stainless steel and none on copper. The variability seen in their experiment is also compatible with the survivability of the SARS-CoV-2 virus on various surfaces.

### B. Effect of surfaces and size of droplets

**FIG. 14.**
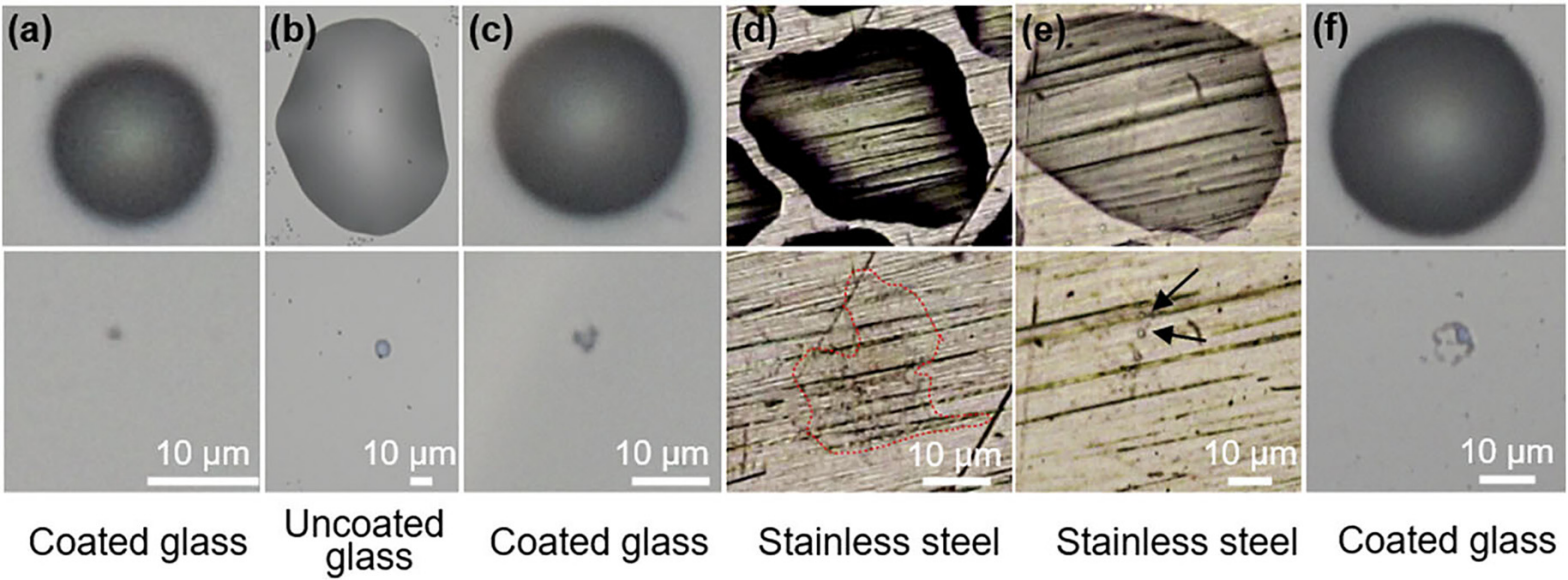
A comparison of the morphologies of the original droplets (top row) and their corresponding residues (bottom row) on different surfaces. Reproduced with the permission from He *et al.*, “Droplet evaporation residue indicating SARS-CoV-2 survivability on surfaces,” Phys. Fluids **33**(1), 013309 (2021).^79^ Copyright 2021AIP Publishing.

**FIG. 15.**
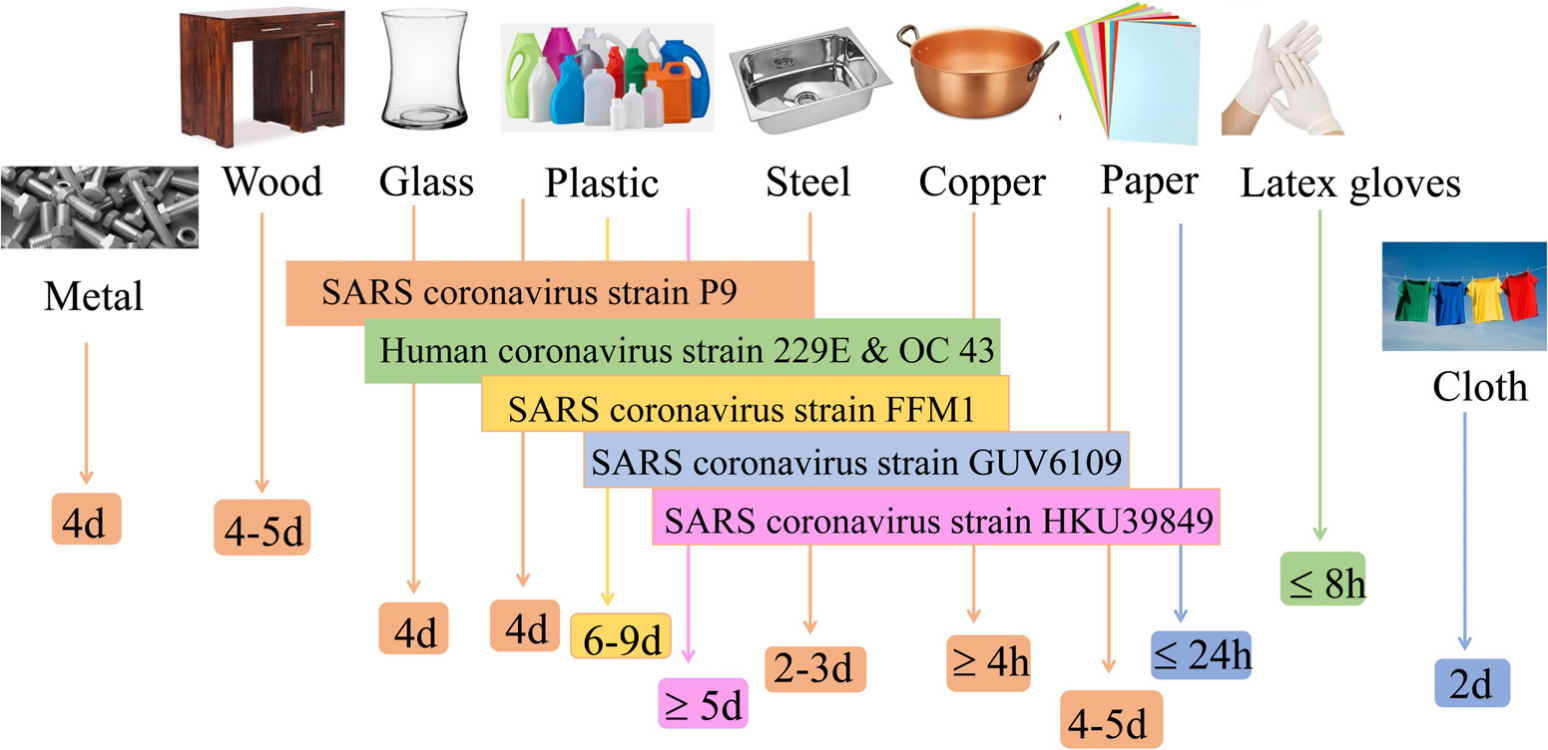
The survival time of various viruses on different surfaces.^44,80^

During evaporation, droplets first shrink in height and then diameter leaving behind the residue as illustrated in Fig. 14. It can be seen that due to evaporation single residues form on glass surfaces with and without hydrophobic coating [Figs. 14(a) and 14(b)]. Figure 14(c) demonstrates the recoiling near the end of evaporation leaving behind a more concentrated residue in the middle of a coated glass substrate due to the influence of surface tension. On strong hydrophilic surfaces, like in the case of a stainless steel surface, the evaporation results in a vast region of thin-film residue [Fig. 14(d)]. Figures 14(e) and 14(f) show the formation of multiple patches of residue due to the pinned film breakage caused by surface roughness and surface tension-induced instabilities, respectively. The infectivity of different strains on various surfaces is illustrated in Fig. 15.^44^ When compared to plastic and glass surfaces, coronavirus strains have a significantly shorter lifetime on metal surfaces. Surfaces with low thermal conductivity (e.g., glass, steel, and plastic) leave residue from varying percentages of all deposited droplets, whereas high heat conductivity surfaces (e.g., copper) leave no resolvable residue.^81^ While Chin *et al.*^82^ found that the SARS-CoV-2 virus is more stable on smooth surfaces (such as glass and plastic) and can survive for two to four days at 60% RH, according to van Doremalen *et al.,*^83^ the virus's lifetime at 40% RH varies from 7 h on copper surfaces to more than three days on plastic surfaces (polypropylene). No contagious virus was found on paper, wood, glass, and banknotes after 3 h, 2, 4, or 7 days, respectively. On plastic materials, the SARS coronavirus strain FFM1 was found to exhibit the greatest lifespan.^44^

**FIG. 16.**
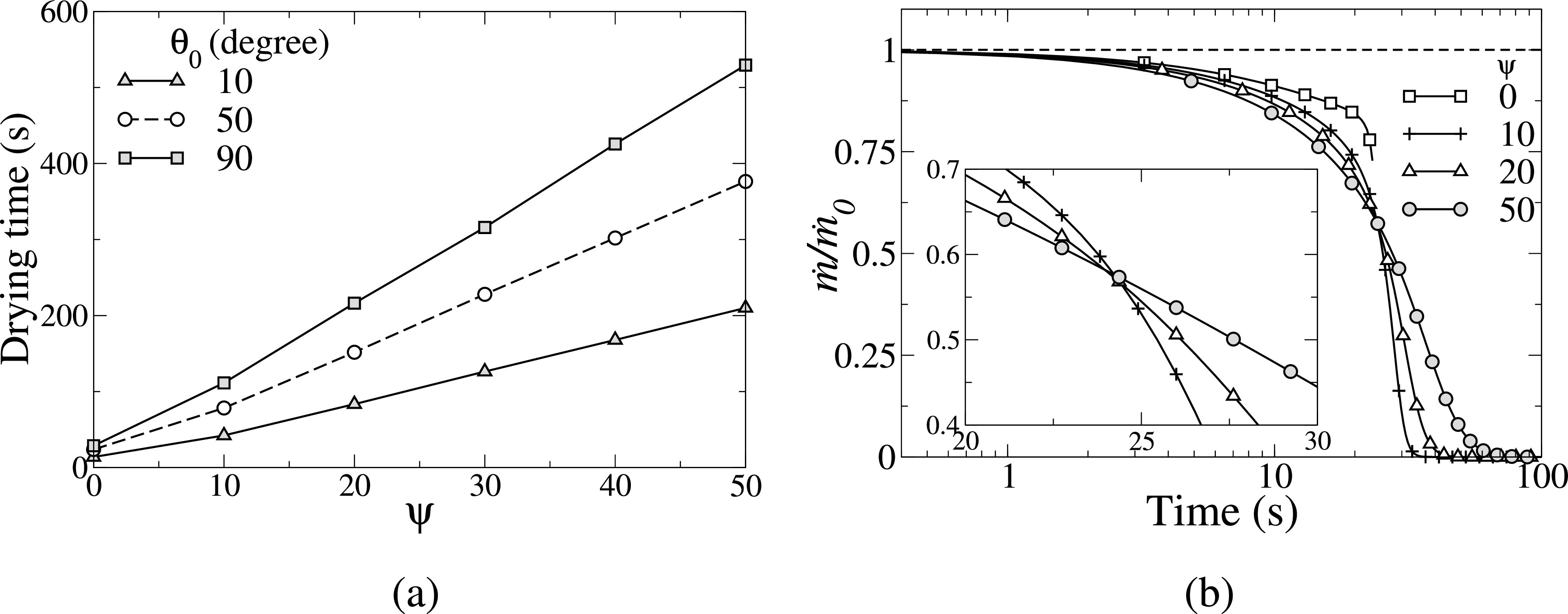
(a) Variation in the droplet drying time with Ψ at different values of the initial contact angle, *θ*_0_. The rest of the parameters are initial volume, *V*_0_ = 10 nL, T = 30 °C, and RH = 50%. (b) The variation of the normalized mass evaporation rate with the initial mass evaporation rate, $\dot{m}/{\dot{m}}_{0}$ vs Ψ. The rest of the parameters are $T=30^{{}^{\circ}}$C, RH = 50% and $\theta_{0}=50^{{}^{\circ}}$. Reproduced with permission from S. Balusamy, S. Banerjee, and K. C. Sahu, “Lifetime of sessile saliva droplets in the context of SARS-CoV-2,” Int. Commun. Heat Mass Transfer. **123**, 105178 (2021).^74^ Copyright 2021 Elsevier.

The physical mechanism associated with survival times of the virus can be understood by studying the evaporation dynamics of these droplets on different surfaces. Some authors^63,73,74^ have used the diffusion-based modeling (discussed in Sec. III A 1) and studied the effect of various surfaces owing to different contact angles, ambient temperature, and relative humidity. The effect of the composition of saliva droplets, [i.e., the presence of salt, protein (mucin), and surfactant (dipalmitoylphosphatidylcholine)] on the evaporation rate and the residue formation was also investigated by Balusamy *et al.*^74^ It was found that a droplet can survive longer on hydrophobic surfaces as compared to a hydrophilic surface and that its lifetime increases as the contact angle increases. Also increasing the concentration of the ingredients (salt, surfactant, protein, etc.) in water droplet [mathematically modeled using Ψ in Eq. (11)] increases the lifetime of the droplet [see Figs. 16(a) and 16(b)]. The three unique zones depicted in Fig. 16(b) are an early period of steady decline, an intermediate period of steep decline, and finally a near-horizontal line at very small values till the end of the droplet lifetime. The final stage is essentially the residual stage, which takes a long time to evaporate.

In order to understand the mechanism of the residue formation at the final stage of evaporation, He *et al.*^79^ proposed a modified physical model that includes the effects of both nonvolatile solute and substrate conductivity on the quasi-steady evaporation rate,

$$\dot{m}=-\pi RD(T)C_{\mathrm{sat}}(T)\left(1-\frac{\phi (t)R_{0}^{3}}{R^{3}}-\text{RH}\right)f(A)\alpha ,$$ (12)

where *R*_0_ is the initial radius of the sessile droplet and ϕ(t) denotes the instantaneous volume fraction of the solute (evaluated by the Nernst and Brunner equation). Here, *α* is associated with the thermal conductivity of the surface. For highly conducting surfaces, like copper, α≈1 and its value decreases with decreasing the thermal conductivity of the surface. They vary the values of *α* from 0.267–1.

He *et al.*^79^ found that the residues observed in their experiments are hard enough even when exposed to changes in ambient temperature and humidity. They can stay on plastic and glass surfaces after being heated for 60 s at 60 °C, although the same treatment removes more than 90% of residues from stainless steel, perhaps because of its greater heat conductivity. The fraction of residue producing droplets increases with increasing the humidity from 25% RH to 60% RH; in coated glass, it grows from 55% to 90%, in plastic, it increases from 5% to 30%, and in copper, it enhances from 0% to 15% (i.e., no residues to residues at higher humidity). Such droplet evaporation-related mechanisms could be key in understanding SARS-CoV-2 carriage and transmission.^84^

### C. Effect of environment condition

**FIG. 17.**
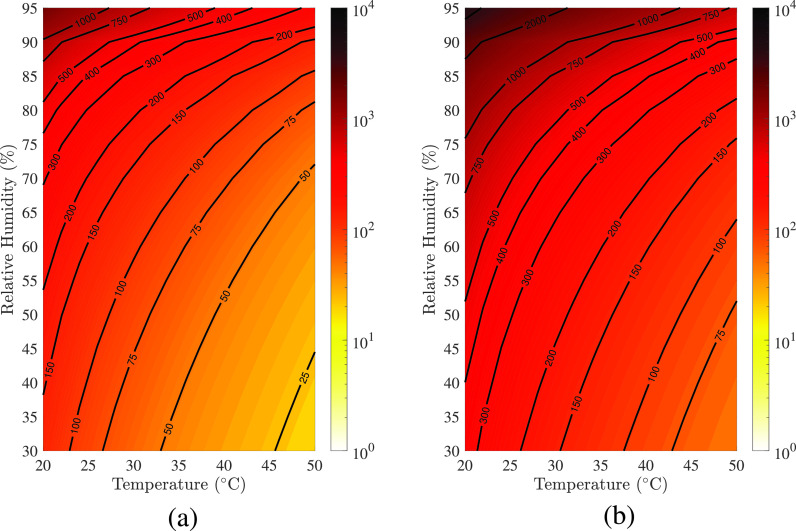
(a) Regime maps depicting the droplet's lifetime in the T− RH space. (a) $\theta_{0}=10^{{}^{\circ}}$, (b) $\theta_{0}=90^{{}^{\circ}}$. The colorbar represents the lifetime in second in the logarithmic scale. The rest of the parameters are $V_{0}=10$ nl, *M *=* *0.154 mol/kg and Ψ=20. Reproduced with permission from S. Balusamy, S. Banerjee, and K. C. Sahu, “Lifetime of sessile saliva droplets in the context of SARS-CoV-2,” Int. Commun. Heat Mass Transfer **123**, 105178 (2021).^74^ Copyright 2021 Elsevier.

Next, we present how environmental conditions, such as ambient temperature and humidity, influence the lifetime of sessile respiratory droplets. Balusamy *et al.*^74^ presented the regime maps depicting the lifetime of a typical saliva droplet ($V_{0}=10$ nl, *M* = 0.154 mol/kg and Ψ=20) on surfaces with contact angles $10^{{}^{\circ}}$ and $90^{{}^{\circ}}$ (see Fig. 17). In these maps, the isocontour lines represent the drying time in seconds. The colormap uses a logarithmic scale to indicate the droplet's drying time, which varies by four orders of magnitude. Two observations can be made from Fig. 17. (i) In a low ambient temperature and humid environment, a droplet takes longer to evaporate, and its lifetime decreases as the humidity decreases and the temperature increases for any surfaces, and (ii) increasing the initial contact angle increases the drying time for a fixed initial droplet volume. This result follows our intuition because a droplet with a smaller contact angle has a bigger wetting radius and surface area for the same volume, allowing for a larger interface area for diffusion-driven evaporation. Therefore, in comparison to less hydrophilic surfaces, highly hydrophilic surfaces may be less sensitive to prolonged contamination. Chen *et al.*^85^ found that the droplet's lifetime decreases with the ambient temperature only when the relative humidity (RH) is less than 37%. When the relative humidity is greater than 37%, raising the ambient temperature prolongs the lifetime of a droplet of the same initial size. The threshold humidity, according to this study, is 55.7% at 30 °C, over which the droplet's lifetime grows exponentially. Bhardwaj *et al.*^63^ also reported that the risk of the virus surviving in a humid environment increases around fivefold when compared to a dry environment.

### D. Indoor vs outdoor transmission

When the air is cooled and dehumidified in an air-conditioned environment, the drying potential is higher than in hot and humid outdoor conditions, resulting in a reduction in the size of the droplets in proportion to the room temperature and relative humidity.^86^ In comparison to hot and humid outdoor conditions, the virus is active due to drying in the cold and humid environment. Even in an outdoor context, a site with little humidity has a greater spread potential. The combined high heat and mass transfer potential of respiratory droplets in low-humidity and low-temperature environments causes quick drying and size reduction, and the virus is almost active everywhere. In a hot and dry region, using a cooling and dehumidification system enhances the drying potential and hence the viral activity to dangerous levels. Thus, using a spray washer to improve cooling and humidification can lower the danger of virus contamination.

### E. Concluding remarks

In this review, we attempt to summarize fluid dynamics studies undertaken concerning coronavirus (SARS-CoV-2) transmission. Several researchers have examined theoretically, numerically, and experimentally both airborne and surfaceborne transmissions through the dispersion and deposition of ejected respiratory droplets, which are the primary sources of COVID-19 propagation. The transmission of respiratory droplets varies based on the situation and is influenced by a variety of factors including ambient conditions, surface properties, social distancing, the use of various kinds of masks, and indoor and outdoor conditions. All of these aspects have been covered in this review. We believe that this study will not only provide some useful guidelines to control the transmission but also benefit researchers in getting a comprehensive assessment of previous work (rapid advancement made by the fluid mechanics community in a short time) in this area and moving forward from the perspective of the fluid dynamics. Despite the extensive set of studies conducted in this area, some key areas where future research may yield valuable insight include (a) experimental studies on tracing droplet particle trajectories over longer distances to complement the current CFD modeling work; (b) modeling of more realistic droplet compositions through treating the ejected droplets as multi-component nano-fluids containing viral nanoparticles; (c) further experimental and modeling work on the impact of multiple coughing or sneezing events in quick succession on the transmissibility of ejected droplets in the exhaled clouds; (d) modeling of not only evaporation but also condensation and freezing on emitted droplets when the ambient temperatures are below the dew point or freeze point temperatures (as in temperate latitude winters). Furthermore, with the emergence of new SARS-CoV-2 variants that are reported to have much higher viral loads,^87^ the modeling assumptions regarding what is the expected virus concentration in the ejected respiratory particles may need to be reviewed with the corresponding impact on determining safe social distances and mask effectiveness.

## Data Availability

The data that support the findings of this study are available from the corresponding authors upon reasonable request.

## References

[1] K. A. Prather, C. C. Wang, and R. T. Schooley, “ Reducing transmission of SARS-CoV-2,” Science 368(6498), 1422–1424 (2020). 10.1126/science.abc6197 32461212

[2] M. Jayaweera, H. Perera, B. Gunawardana, and J. Manatunge, “ Transmission of COVID-19 virus by droplets and aerosols: A critical review on the unresolved dichotomy,” Environ. Res 188, 109819 (2020). 10.1016/j.envres.2020.109819 32569870 PMC7293495

[3] D. A. Edwards, J. C. Man, P. Brand, J. P. Katstra, K. Sommerer, H. A. Stone, E. Nardell, and G. Scheuch, “ Inhaling to mitigate exhaled bioaerosols,” Proc. Natl. Acad. Sci. U. S. A. 101(50), 17383–17388 (2004). 10.1073/pnas.0408159101 15583121 PMC536048

[4] E. P. Vejerano and L. C. Marr, “ Physico-chemical characteristics of evaporating respiratory fluid droplets,” J. R. Soc. Interface 15(139), 20170939 (2018). 10.1098/rsif.2017.0939 29491178 PMC5832737

[5] A. H. Shafaghi, F. Rokhsar Talabazar, A. Koşar, and M. Ghorbani, “ on the effect of the respiratory droplet generation condition on COVID-19 transmission,” Fluids 5(3), 113 (2020). 10.3390/fluids5030113

[6] R. Mittal, R. Ni, and J.-H. Seo, “ The flow physics of COVID-19,” J. Fluid Mech. 894, F2 (2020).

[7] R. R. Netz, “ Mechanisms of airborne infection via evaporating and sedimenting droplets produced by speaking,” J. Phys. Chem. B 124(33), 7093–7101 (2020). 10.1021/acs.jpcb.0c05229 32668904 PMC7409921

[8] S. K. Das, J. E. Alam, S. Plumari, and V. Greco, “ Transmission of airborne virus through sneezed and coughed droplets,” Phys. Fluids 32(9), 097102 (2020). 10.1063/5.0022859 PMC7513825 32982136

[9] R. Singhal, S. Ravichandran, R. Govindarajan, and S. S. Diwan, “ Virus transmission by aerosol transport during short conversations,” preprint arXiv:2103.16415 (2021).

[10] M. Abkarian, S. Mendez, N. Xue, F. Yang, and H. A. Stone, “ Speech can produce jet-like transport relevant to asymptomatic spreading of virus,” Proc. Natl. Acad. Sci. U. S. A. 117(41), 25237–25245 (2020). 10.1073/pnas.2012156117 32978297 PMC7568291

[11] S. Balachandar, S. Zaleski, A. Soldati, G. Ahmadi, and L. Bourouiba, “ Host-to-host airborne transmission as a multiphase flow problem for science-based social distance guidelines,” Int. J. Multiphase Flow 132, 103439 (2020).

[12] Y. Ma, Y. Zhao, J. Liu, X. He, B. Wang, S. Fu, J. Yan, J. Niu, J. Zhou, and B. Luo, “ Effects of temperature variation and humidity on the death of COVID-19 in Wuhan, China,” Sci. Total Environ. 724, 138226 (2020). 10.1016/j.scitotenv.2020.138226 32408453 PMC7142681

[13] R. K. Bhagat, M. D. Wykes, S. B. Dalziel, and P. Linden, “ Effects of ventilation on the indoor spread of COVID-19,” J. Fluid Mech. 903, F1 (2020). 10.1017/jfm.2020.720 34191877 PMC7520710

[14] L. Morawska and J. Cao, “ Airborne transmission of SARS-CoV-2: The world should face the reality,” Environ. Int 139, 105730 (2020). 10.1016/j.envint.2020.105730 32294574 PMC7151430

[15] L. Morawska and D. K. Milton, “ It is time to address airborne transmission of coronavirus disease 2019 (COVID-19,” Clin. Infect. Dis 71(9), 2311–2313 (2020). 10.1093/cid/ciaa939 32628269 PMC7454469

[16] G. Scheuch, “ Breathing is enough: For the spread of influenza virus and SARS-CoV-2 by breathing only,” J. Aerosol Med. Pulm. Drug Delivery 33(4), 230–234 (2020). 10.1089/jamp.2020.1616 PMC7406993 32552296

[17] P. Bahl, C. Doolan, C. De Silva, A. A. Chughtai, L. Bourouiba, and C. R. MacIntyre, “ Airborne or droplet precautions for health workers treating COVID-19?,” J. Infect. Dis. 2020, jiaa189. 10.1093/infdis/jiaa189 PMC7184471 32301491

[18] L. Setti, F. Passarini, G. De Gennaro, P. Barbieri, M. G. Perrone, M. Borelli, J. Palmisani, A. D. Gilio, P. Piscitelli, and A. Miani, “ Airborne transmission route of COVID-19: Why 2 meters/6 feet of inter-personal distance could not be enough,” Int. J. Environ. Res. Public Health 17(8), 2932 (2020). 10.3390/ijerph17082932 PMC7215485 32340347

[19] F. Yang, A. A. Pahlavan, S. Mendez, M. Abkarian, and H. A. Stone, “ Towards improved social distancing guidelines: Space and time dependence of virus transmission from speech-driven aerosol transport between two individuals,” Phys. Rev. Fluids 5(12), 122501 (2020). 10.1103/PhysRevFluids.5.122501

[20] M. Hossain and N. H. Faisal, “ Modeling aerosol cloud aerodynamics during human coughing, talking, and breathing actions,” AIP Adv. 11(4), 045111 (2021). 10.1063/5.0042952

[21] X. Xie, Y. Li, A. Chwang, P. Ho, and W. Seto, “ How far droplets can move in indoor environments–revisiting the wells evaporation-falling curve,” Indoor Air 17(3), 211–225 (2007). 10.1111/j.1600-0668.2007.00469.x 17542834

[22] J. Duguid, “ The size and the duration of air-carriage of respiratory droplets and droplet-nuclei,” Epidemiol. Infect. 44(6), 471–479 (1946). 10.1017/S0022172400019288 PMC2234804 20475760

[23] C. Y. H. Chao, M. P. Wan, L. Morawska, G. R. Johnson, Z. D. Ristovski, M. Hargreaves, K. Mengersen, S. Corbett, Y. Li, X. Xie, and D. Katoshevskig, “ Characterization of expiration air jets and droplet size distributions immediately at the mouth opening,” J. Aerosol Sci. 40(2), 122–133 (2009). 10.1016/j.jaerosci.2008.10.003 32287373 PMC7126899

[24] R. G. Loudon and R. M. Roberts, “ Droplet expulsion from the respiratory tract,” Am. J. Respir. Crit. Care Med. 95(3), 435–442 (1967). 10.1164/arrd.1967.95.3.435 6018703

[25] L. Morawska, G. Johnson, Z. Ristovski, M. Hargreaves, K. Mengersen, S. Corbett, C. Y. H. Chao, Y. Li, and D. Katoshevski, “ Size distribution and sites of origin of droplets expelled from the human respiratory tract during expiratory activities,” J. Aerosol Sci. 40(3), 256–269 (2009). 10.1016/j.jaerosci.2008.11.002 PMC7126899 32287373

[26] L. Bourouiba, E. Dehandschoewercker, and J. W. Bush, “ Violent expiratory events: On coughing and sneezing,” J. Fluid Mech. 745, 537–563 (2014). 10.1017/jfm.2014.88

[27] T. Dbouk and D. Drikakis, “ On coughing and airborne droplet transmission to humans,” Phys. Fluids 32(5), 053310 (2020). 10.1063/5.0011960 PMC7239332 32574229

[28] A. Agrawal and R. Bhardwaj, “ Reducing chances of COVID-19 infection by a cough cloud in a closed space,” Phys. Fluids 32(10), 101704 (2020). 10.1063/5.0029186 PMC7583278 33100805

[29] L. Bourouiba, “ Turbulent gas clouds and respiratory pathogen emissions: Potential implications for reducing transmission of COVID-19,” JAMA 323(18), 1837–1838 (2020). 10.1001/jama.2020.4756 32215590

[30] C. P. Cummins, O. J. Ajayi, F. V. Mehendale, R. Gabl, and I. M. Viola, “ The dispersion of spherical droplets in source–sink flows and their relevance to the COVID-19 pandemic,” Phys. Fluids 32(8), 083302 (2020). 10.1063/5.0021427 PMC7437950 32831537

[31] S. S. Diwan, S. Ravichandran, R. Govindarajan, and R. Narasimha, “ Understanding transmission dynamics of COVID-19-type infections by direct numerical simulations of cough/sneeze flows,” Trans. Indian Natl. Acad. Eng. 5, 255–261 (2020). 10.1007/s41403-020-00106-w PMC7268977 38624374

[32] H. Li, F. Y. Leong, G. Xu, Z. Ge, C. W. Kang, and K. H. Lim, “ Dispersion of evaporating cough droplets in tropical outdoor environment,” Phys. Fluids 32(11), 113301 (2020). 10.1063/5.0026360 PMC7685245 33244215

[33] Y. Feng, T. Marchal, T. Sperry, and H. Yi, “ Influence of wind and relative humidity on the social distancing effectiveness to prevent COVID-19 airborne transmission: A numerical study,” J. Aerosol Sci. 147, 105585 (2020). 10.1016/j.jaerosci.2020.105585 32427227 PMC7233256

[34] R. Mittal, C. Meneveau, and W. Wu, “ A mathematical framework for estimating risk of airborne transmission of COVID-19 with application to face mask use and social distancing,” Phys. Fluids 32(10), 101903 (2020). 10.1063/5.0025476 PMC7583361 33100806

[35] L. Zhao, Y. Qi, P. Luzzatto-Fegiz, Y. Cui, and Y. Zhu, “ COVID-19: Effects of environmental conditions on the propagation of respiratory droplets,” Nano Lett. 20(10), 7744–7750 (2020). 10.1021/acs.nanolett.0c03331 32909761

[36] Y. M. Bar-On, A. Flamholz, R. Phillips, R. Milo, and f Science, “ SARS-CoV-2 (COVID-19) by the numbers,” eLife 9, e57309 (2020). 10.7554/eLife.57309 32228860 PMC7224694

[37] S. Kumar, “ Effect of meteorological parameters on spread of COVID-19 in India and air quality during lockdown,” Sci. Total Environ. 745, 141021 (2020). 10.1016/j.scitotenv.2020.141021 32702548 PMC7369006

[38] C. Sun and Z. Zhai, “ The efficacy of social distance and ventilation effectiveness in preventing COVID-19 transmission,” Sustainable Cities Soc. 62, 102390 (2020). 10.1016/j.scs.2020.102390 PMC7357531 32834937

[39] C. D. Zangmeister, J. G. Radney, E. P. Vicenzi, and J. L. Weaver, “ Filtration efficiencies of nanoscale aerosol by cloth mask materials used to slow the spread of SARS-CoV-2,” ACS Nano 14(7), 9188–9200 (2020). 10.1021/acsnano.0c05025 32584542

[40] J. Akhtar, A. L. Garcia, L. Saenz, S. Kuravi, F. Shu, and K. Kota, “ Can face masks offer protection from airborne sneeze and cough droplets in close-up, face–to–face human interactions?–A quantitative study,” Phys. Fluids 32(12), 127112 (2020). 10.1063/5.0035072 PMC7757609 33362404

[41] A. Khosronejad, C. Santoni, K. Flora, Z. Zhang, S. Kang, S. Payabvash, and F. Sotiropoulos, “ Fluid dynamics simulations show that facial masks can suppress the spread of COVID-19 in indoor environments,” AIP Adv. 10(12), 125109 (2020). 10.1063/5.0035414

[42] J. Wei, S. Guo, E. Long, L. Zhang, B. Shu, and L. Guo, “ Why does the spread of COVID-19 vary greatly in different countries? Revealing the efficacy of face masks in epidemic prevention,” Epidemiol. Infect. 149, e24 (2021). 33441205 10.1017/S0950268821000108 PMC7844184

[43] T. Dbouk and D. Drikakis, “ On respiratory droplets and face masks,” Phys. Fluids 32(6), 063303 (2020). 10.1063/5.0015044 PMC7301882 32574231

[44] G. Seminara, B. Carli, G. Forni, S. Fuzzi, A. Mazzino, and A. Rinaldo, “ Biological fluid dynamics of airborne COVID-19 infection,” Rend. Lincei Sci. Fis. Nat. 2020, 1–33. 10.1007/s12210-020-00938-2 PMC7429142 32837713

[45] E. Atangana and A. Atangana, “ Facemasks simple but powerful weapons to protect against COVID-19 spread: Can they have sides effects?,” Results Phys. 19, 103425 (2020). 10.1016/j.rinp.2020.103425 33014697 PMC7525365

[46] J. Panovska-Griffiths, C. C. Kerr, W. Waites, R. M. Stuart, D. Mistry, D. Foster, D. J. Klein, R. M. Viner, and C. Bonell, “ Modelling the potential impact of mask use in schools and society on COVID-19 control in the UK,” Sci. Rep. 11(1), 1–12 (2021). 10.1038/s41598-021-88075-0 33888818 PMC8062670

[47] A. Foster and M. Kinzel, “ Estimating COVID-19 exposure in a classroom setting: A comparison between mathematical and numerical models,” Phys. Fluids 33(2), 021904 (2021). 10.1063/5.0040755 PMC7975712 33746487

[48] J. Lu, J. Gu, K. Li, C. Xu, W. Su, Z. Lai, D. Zhou, C. Yu, B. Xu, and Z. Yang, “ COVID-19 outbreak associated with air conditioning in restaurant, Guangzhou, China, 2020,” Emerging Infect. Dis. 26(7), 1628 (2020). 10.3201/eid2607.200764 PMC7323555 32240078

[49] Y. Li, H. Qian, J. Hang, X. Chen, P. Cheng, H. Ling, S. Wang, P. Liang, J. Li, S. Xiao *et al.*, “ Probable airborne transmission of SARS-CoV-2 in a poorly ventilated restaurant,” Build. Environ. 196, 107788 (2021). 10.1016/j.buildenv.2021.107788 33746341 PMC7954773

[50] K. L. Chong, C. S. Ng, N. Hori, R. Yang, R. Verzicco, and D. Lohse, “ Extended lifetime of respiratory droplets in a turbulent vapor puff and its implications on airborne disease transmission,” Phys. Rev. Lett. 126(3), 034502 (2021). 10.1103/PhysRevLett.126.034502 33543958

[51] W. F. Wells, “ On air-borne infection. study II. Droplets and droplet nuclei,” Am. J. Hygiene 20, 611–618 (1934).

[52] M. Abuhegazy, K. Talaat, O. Anderoglu, and S. V. Poroseva, “ Numerical investigation of aerosol transport in a classroom with relevance to COVID-19,” Phys. Fluids 32(10), 103311 (2020). 10.1063/5.0029118 PMC7583363 33100808

[53] B. Blocken, T. van Druenen, A. Ricci, L. Kang, T. van Hooff, P. Qin, L. Xia, C. A. Ruiz, J. Arts, J. Diepens *et al.*, “ Ventilation and air cleaning to limit aerosol particle concentrations in a gym during the COVID-19 pandemic,” Build. Environ. 193, 107659 (2021). 10.1016/j.buildenv.2021.107659 33568882 PMC7860965

[54] A. Pavansai, P. Deepak, S. R. Hari, R. Harish, and M. S. Kumar, “ Analyzing social distancing policy effectiveness using computational fluid dynamics inside a bus to prevent COVID-19 airborne transmission,” in *IOP Conference Series: Materials Science and Engineering* ( IOP Publishing, 2021), Vol. 1128, p. 012005.

[55] V. Mathai, A. Das, J. A. Bailey, and K. Breuer, “ Airflows inside passenger cars and implications for airborne disease transmission,” Sci. Adv. 7(1), eabe0166 (2021). 10.1126/sciadv.abe0166 33277325 PMC7775778

[56] N. Sen, “ Transmission and evaporation of cough droplets in an elevator: Numerical simulations of some possible scenarios,” Phys. Fluids 33(3), 033311 (2021). 10.1063/5.0039559 PMC7976048 33746494

[57] J. A. Adeniran, I. A. Mohammed, O. I. Muniru, T. Oloyede, O. O. Sonibare, M.-N. O. Yusuf, K. A. Abdulraheem, E. T. Odediran, R. O. Yusuf, and J. A. Sonibare, “ Indoor transmission dynamics of expired SARS-CoV-2 virus in a model African hospital ward,” J. Environ. Health Sci. Eng. 19(7), 331–341 (2021). 10.1007/s40201-020-00606-5 PMC7821173 33500782

[58] S.-J. Jang, S.-S. Baek, J.-Y. Kim, and S.-H. Hwang, “ Preparation and adhesion performance of transparent acrylic pressure sensitive adhesives for touch screen panel,” J. Adhes. Sci. Technol. 28(19), 1990–2000 (2014). 10.1080/01694243.2014.940664

[59] Y. L. Hsieh, J. Thompson, and A. Miller, “ Water wetting and retention of cotton assemblies as affected by alkaline and bleaching treatments,” Text. Res. J. 66(7), 456–464 (1996). 10.1177/004051759606600707

[60] S. Chandra and C. T. Avedisian, “ On the collision of a droplet with a solid surface,” Proc. R. Soc. A 432(1991), 13–41 (1884).

[61] G. I. Mantanis and R. A. Young, “ Wetting of wood,” Wood Sci. Technol. 31(5), 339 (1997). 10.1007/BF01159153

[62] D. C. D. Roux and J. J. Cooper-White, “ Dynamics of water spreading on a glass surface,” J. Colloid Interface Sci. 277(2), 424–436 (2004). 10.1016/j.jcis.2004.05.007 15341855

[63] R. Bhardwaj and A. Agrawal, “ Likelihood of survival of coronavirus in a respiratory droplet deposited on a solid surface,” Phys. Fluids 32(6), 061704 (2020). 10.1063/5.0012009 PMC7295365 32574230

[64] G. Karapetsas, K. C. Sahu, and O. K. Matar, “ Effect of contact line dynamics on the thermocapillary motion of a droplet on an inclined plate,” Langmuir 29(28), 8892–8906 (2013). 10.1021/la4014027 23786489

[65] G. Karapetsas, K. C. Sahu, K. Sefiane, and O. K. Matar, “ Thermocapillary-driven motion of a sessile drop: Effect of non-monotonic dependence of surface tension on temperature,” Langmuir 30(15), 4310–4321 (2014). 10.1021/la5002682 24694047

[66] D. Brutin and V. Starov, “ Recent advances in droplet wetting and evaporation,” Chem. Soc. Rev. 47(2), 558–585 (2018). 10.1039/C6CS00902F 29090296

[67] P. Gurrala, P. Katre, S. Balusamy, S. Banerjee, and K. C. Sahu, “ Evaporation of ethanol-water sessile droplet of different compositions at an elevated substrate temperature,” Int. J. Heat Mass Transfer 145, 118770 (2019). 10.1016/j.ijheatmasstransfer.2019.118770

[68] P. Katre, P. Gurrala, S. Balusamy, S. Banerjee, and K. C. Sahu, “ Evaporation of sessile ethanol-water droplets on a critically inclined heated surface,” Int. J. Multiphase Flow 131, 103368 (2020). 10.1016/j.ijmultiphaseflow.2020.103368

[69] P. Katre, S. Balusamy, S. Banerjee, L. D. Chandrala, and K. C. Sahu, “ Evaporation dynamics of a sessile droplet of binary mixture laden with nanoparticles,” Langmuir 37, 6311–6321 (2021). 10.1021/acs.langmuir.1c00806 33983033

[70] P. Gurrala, S. Balusamy, S. Banerjee, and K. C. Sahu, “ A review on the evaporation dynamics of sessile drops of binary mixtures: Challenges and opportunities,” Fluid Dyn. Mater. Process. 17(2), 253–284 (2021). 10.32604/fdmp.2021.014126

[71] G. Karapetsas, K. C. Sahu, and O. K. Matar, “ Evaporation of sessile droplets laden with particles and insoluble surfactants,” Langmuir 32(27), 6871–6881 (2016). 10.1021/acs.langmuir.6b01042 27300638

[72] V. Soulié, S. Karpitschka, F. Lequien, P. Prené, T. Zemb, H. Moehwald, and H. Riegler, “ The evaporation behavior of sessile droplets from aqueous saline solutions,” Phys. Chem. Chem. Phys 17(34), 22296–22303 (2015). 10.1039/C5CP02444G 26246358

[73] R. Bhardwaj and A. Agrawal, “ How coronavirus survives for days on surfaces,” Phys. Fluids 32(11), 111706 (2020). 10.1063/5.0033306 PMC7713872 33281435

[74] S. Balusamy, S. Banerjee, and K. C. Sahu, “ Lifetime of sessile saliva droplets in the context of SARS-CoV-2,” Int. Commun. Heat Mass Transfer 123, 105178 (2021). 10.1016/j.icheatmasstransfer.2021.105178

[75] R. H. Perry, D. W. Green, and J. O. Maloney, *Perry's Chemical Engineers' Handbook*, 8th ed. ( McGrawHill Education, 2008).

[76] Y. Fukatani, D. Orejon, Y. Kita, Y. Takata, J. Kim, and K. Sefiane, “ Effect of ambient temperature and relative humidity on interfacial temperature during early stages of drop evaporation,” Phys. Rev. E 93(4), 043103 (2016). 10.1103/PhysRevE.93.043103 27176386

[77] S. Basu, P. Kabi, S. Chaudhuri, and A. Saha, “ Insights on drying and precipitation dynamics of respiratory droplets from the perspective of COVID-19,” Phys. Fluids 32(12), 123317 (2020). 10.1063/5.0037360 PMC7976039 33746480

[78] C. Lieber, S. Melekidis, R. Koch, and H.-J. Bauer, “ Insights into the evaporation characteristics of saliva droplets and aerosols: Levitation experiments and numerical modeling,” J. Aerosol Sci. 154, 105760 (2021). 10.1016/j.jaerosci.2021.105760 33518792 PMC7826107

[79] Z. He, S. Shao, J. Li, S. S. Kumar, J. B. Sokoloff, and J. Hong, “ Droplet evaporation residue indicating SARS-CoV-2 survivability on surfaces,” Phys. Fluids 33(1), 013309 (2021). 10.1063/5.0038562 PMC7976051 33746482

[80] H. Fathizadeh, P. Maroufi, M. Momen-Heravi, S. Dao, K. Ganbarov, P. Pagliano, S. Esposito, H. S. Kafil *et al.*, “ Protection and disinfection policies against SARS-CoV-2 (COVID-19),” Infez. Med. 28(2), 185–191 (2020). 32275260

[81] R. Suman, M. Javaid, A. Haleem, R. Vaishya, S. Bahl, and D. Nandan, “ Sustainability of coronavirus on different surfaces,” J. Clin. Exp. Hepatol. 10(4), 386–390 (2020). 10.1016/j.jceh.2020.04.020 32377058 PMC7201236

[82] A. W. H. Chin and L. L. M. Poon, “ Stability of SARS-CoV-2 in different environmental conditions–authors' reply,” Lancet Microbe 1, e10 (2020). 10.1016/S2666-5247(20)30003-3 33521712 PMC7833315

[83] N. Van Doremalen, T. Bushmaker, D. H. Morris, M. G. Holbrook, A. Gamble, B. N. Williamson, A. Tamin, J. L. Harcourt, N. J. Thornburg, S. I. Gerber, and J. O. Lloyd-Smith, “ Aerosol and surface stability of SARS-CoV-2 as compared with SARS-CoV-1,” N. Engl. J. Med 382(16), 1564–1567 (2020). 10.1056/NEJMc2004973 32182409 PMC7121658

[84] J. Gralton, E. Tovey, M. L. McLaws, and W. D. Rawlinson, “ The role of particle size in aerosolised pathogen transmission: A review,” J. Infect 62(1), 1–13 (2011). 10.1016/j.jinf.2010.11.010 21094184 PMC7112663

[85] L.-D. Chen, “ Effects of ambient temperature and humidity on droplet lifetime—A perspective of exhalation sneeze droplets with COVID-19 virus transmission,” Int. J. Hyg. Environ. Health 229, 113568 (2020). 10.1016/j.ijheh.2020.113568 32615522 PMC7274593

[86] R. Velraj, F. Haghighat *et al.*, “ The contribution of dry indoor built environment on the spread of coronavirus: Data from various Indian states,” Sustainable Cities Soc. 62, 102371 (2020). 10.1016/j.scs.2020.102371 PMC7329687 32834934

[87] B. Li, A. Deng, K. Li, Y. Hu, Z. Li, Q. Xiong, Z. Liu, Q. Guo, L. Zou, H. Zhang, and M. Zhang, “ Viral infection and transmission in a large well-traced outbreak caused by the delta SARS-CoV-2 variant,” medRxiv (2021). 10.1038/s41467-022-28089-y PMC8786931 35075154

